# Heteroatom Doping Restructures Interfacial H_2_O to Resolve Mechanistic Contradictions in CO_2_ Electroreduction

**DOI:** 10.1021/jacs.6c08293

**Published:** 2026-07-20

**Authors:** Lingyue Liu, Haihui Lan, Li Li, Weijue Wang, Yuhang Jin, Guoqin Liu, Yuhang Liu, Wenqiang Yang, Ming Zhao, Jie Ding, Hongbin Yang, Yanqiang Huang, Xinliang Feng

**Affiliations:** † Max Planck Institute of Microstructure Physics, Halle (Saale) 06120, Germany; ‡ Department of Chemistry, 2167 Massachusetts Institute of Technology, Cambridge, Massachusetts 02139, United States; § State Key Laboratory of Catalysis, 58279 Dalian Institute of Chemical Physics, Chinese Academy of Sciences, Dalian 116023, China; ∥ University of Chinese Academy of Sciences, Beijing 100049, China; ⊥ School of Materials Science and Engineering, 66339 Suzhou University of Science and Technology, Suzhou 215009, China; # Faculty of Chemistry and Food Chemistry & Center for Advancing Electronics Dresden (CFAED), 9169 Technische Universität Dresden, Dresden 01062, Germany

## Abstract

Interfacial H_2_O is increasingly recognized as an active participant in electrocatalysis, yet a central challenge remains: how to deliberately and predictably program its microscopic structure to steer proton-coupled electron transfer (PCET) and selectivity. Here we introduce nonmetal doping as a materials-encoded handle to tune interfacial hydration on a model SnO_2_ catalyst without changing its bulk phase. Across N, P and S dopants, S uniquely stabilizes a disordered, predominantly H-down hydration motif at the catalyst-electrolyte boundary, which promotes H_2_O activation and directional proton delivery, accelerating PCET toward formate formation. Notably, this enhancement arises despite weaker binding of CO_2_-derived intermediates, revealing a solution-structure-controlled selectivity lever that can override conventional binding-energy-based expectations. By establishing a causal link between a doping-defined hydration motif and reaction kinetics, this work elevates interfacial H_2_O from a “context” to a designable variable, and suggests a broadly applicable strategy for optimizing PCET-governed transformations via interfacial solvation engineering.

## Graphical abstract

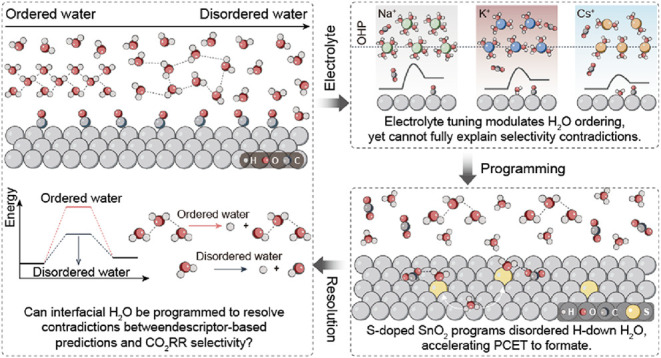

## Introduction

Electrocatalytic reactions occur at the delicate interface between catalyst surfaces and electrolyte solvents, where the structure and dynamics of interfacial H_2_O layers have long been regarded as spectators.^1–3^ Yet recent studies have begun to unravel a more active role of these H_2_O layers in shaping reactivity and selectivity.^4–6^ In the case of carbon dioxide electroreduction (CO_2_RR), while catalyst composition and intermediate adsorption energies have traditionally been considered the primary descriptors for product selectivity, growing evidence suggests that interfacial solvation structure can tip the balance between competing pathways.^7–9^

A key conceptual shift is that interfacial H_2_O is not merely a background medium: ordered hydration layers stabilized by extended hydrogen-bond networks can impede elementary proton-coupled electron transfer (PCET), whereas disordered hydration, particularly with a high H-down population, may facilitate PCET-driven pathways (Figure 1a).^10–15^ Yet, mechanistic interpretations of CO_2_RR still predominantly rely on static surface descriptors such as intermediate binding energies or metal oxidation states, which often fail to reconcile predicted trends with experimentally observed selectivity. This mismatch points to interfacial solvation, and especially the microscopic structure of interfacial H_2_O, as an independent variable that must be considered alongside adsorbate energetics. Electrolyte- and additive-driven microenvironment regulation has provided compelling precedents, for example, engineering cation solvation structures or interfacial hydrogen-bond networks can reshape local proton availability and reaction barriers (Figure 1b); however, these approaches predominantly rely on electrolyte-side tuning or external modifiers, whereas catalyst-side hydration programming encoded by the catalyst lattice itself remains far less explored.^16–21^ These studies collectively support a view in which H_2_O can actively modulate transition-state stabilization depending on its orientation and degree of order.^22,23^ However, compared with electrolyte-side solvation engineering or adsorbate/additive-mediated interfacial restructuring, a dopant-identity-defined, catalyst-encoded strategy that stabilizes a measurable hydration motif (connectivity-disrupted yet orientation-biased, H-down-rich) and links it quantitatively to PCET-controlled CO_2_RR kinetics is still underdeveloped, motivating the present study.^24,25^ We hypothesize that heteroatom doping offers a practical route to encode local electrostatics and hydrogen-bonding propensity at the surface, thereby stabilizing distinct hydration motifs (including disordered, H-down-rich domains) that are favorable for PCET (Figure 1c).^26,27^

**Figure 1:**
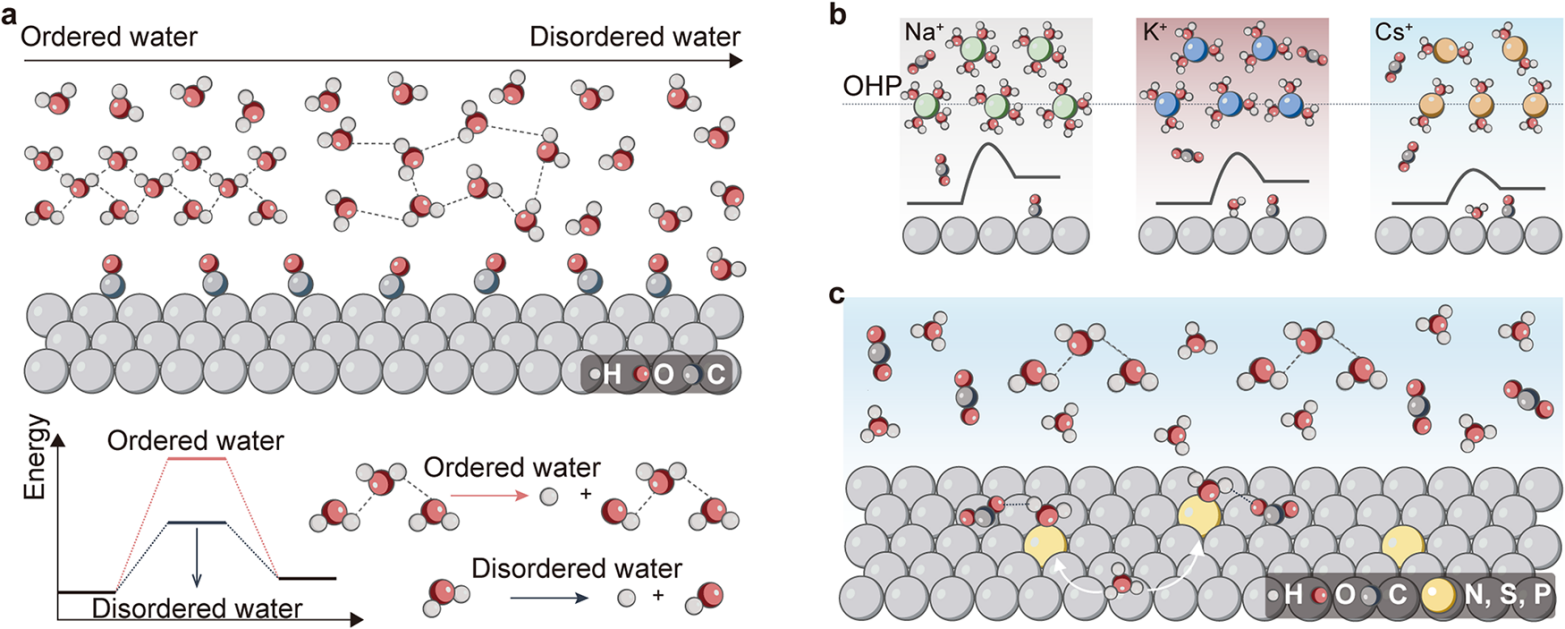
Modulating interfacial H_2_O structure through nonmetal doping in electrocatalysis. (a) Schematic illustration showing the transition from ordered to disordered H_2_O layers at the catalyst-electrolyte interface. While ordered H_2_O forms a robust hydrogen-bond network that stabilizes water molecules, disordered H_2_O exhibits enhanced molecular dynamics, facilitating H_2_O dissociation and proton-coupled electron transfer (PCET) by lowering the activation energy barrier. (b) Representative strategy of regulating interfacial H_2_O structure using hydrated alkali metal cations (Na^+^, K^+^, Cs^+^). Varying cation hydration energies and sizes affect the outer Helmholtz plane and influence H_2_O orientation and dissociation energetics. (c) Conceptual design of nonmetal heteroatom doping (N, S, P) into the catalyst lattice to directly perturb the interfacial H_2_O network. Beyond electronic modulation of the active site, these heteroatoms serve as polar interaction centers that restructure the local hydrogen-bonding environment, thereby promoting interfacial H_2_O disordering and lowering the barrier for H_2_O activation in electrocatalytic reactions.

Here, using S-doped SnO_2_ (S-SnO_2_) as a model system, we show that heteroatom-modified surfaces can stabilize a unique population of disordered interfacial H_2_O with predominant H-down orientation, as supported by AIMD simulations and *operando* spectroscopic signatures. More importantly, we establish a testable mechanistic framework in which a doping-encoded hydration motif regulates H_2_O activation and directional proton delivery, thereby setting the effective barrier for the CO_2_-to-formate pathway beyond conventional binding-energy descriptors. Combining *operando* measurements, AIMD-derived hydration statistics, isotopic labeling and kinetic isotope effect analyses, we demonstrate that the microstructural H_2_O environment can dictate CO_2_RR selectivity even when CO_2_-derived intermediates bind more weakly. This framework provides a falsifiable prediction and a broadly applicable design handle for PCET-governed electrocatalysis: programming interfacial solvation can rationalize and potentially overturn selectivity trends inferred solely from adsorbate-binding energetics.

## Results and Discussion

To establish a model platform for dissecting the role of interfacial water structure in CO_2_ electroreduction, we synthesized a family of nonmetal-doped SnO_2_ catalysts via a gas–solid interface annealing strategy (Figure S1). SnO_2_ nanoparticles were exposed to vapor-phase precursors of urea (N source), thiourea (S source), and sodium hypophosphite (P source) under an Ar atmosphere, with temperatures of 260 °C on the dopant side and 200 °C on the SnO_2_ side. This setup enabled directional diffusion of heteroatoms across the interface and their subsequent incorporation into the SnO_2_ lattice. The resulting materials, denoted as N-SnO_2_, P-SnO_2_, and S-SnO_2_, retained the crystalline phase and morphology of pristine SnO_2_, as confirmed by powder X-ray diffraction (PXRD) (Figure S2), transmission electron microscopy (TEM, Figure S3), and elemental mapping (Figure S4). X-ray photoelectron spectroscopy (XPS) confirmed the presence of Sn–N, Sn–P, and Sn–S coordination environments (Figure 2a), alongside residual N–O, P–O, and S–O species, indicating successful dopant incorporation rather than surface physisorption. Subtle shifts in Sn 3d binding energies (Figure S5a) and increased O 1s binding energies (Figure S5b) in doped samples suggest altered Sn electronic environments and enhanced electron withdrawal from lattice oxygen. To quantify the dopant-induced electronic perturbation, we performed Sn K-edge X-ray absorption near-edge structure (XANES) spectroscopy (Figure 2b).^28^ All doped samples exhibit blue-shifted edge positions relative to pristine SnO_2_, indicative of increased Sn oxidation states. Among them, P-SnO_2_ displays the largest shift, implying the strongest electron-withdrawing effect. Linear combination fitting (Figure 2b, inset) confirms the valence order: P-SnO_2_ > S-SnO_2_ > N-SnO_2_ > SnO_2_, highlighting the sensitivity of Sn electronic structure to the dopant identity.

**Figure 2:**
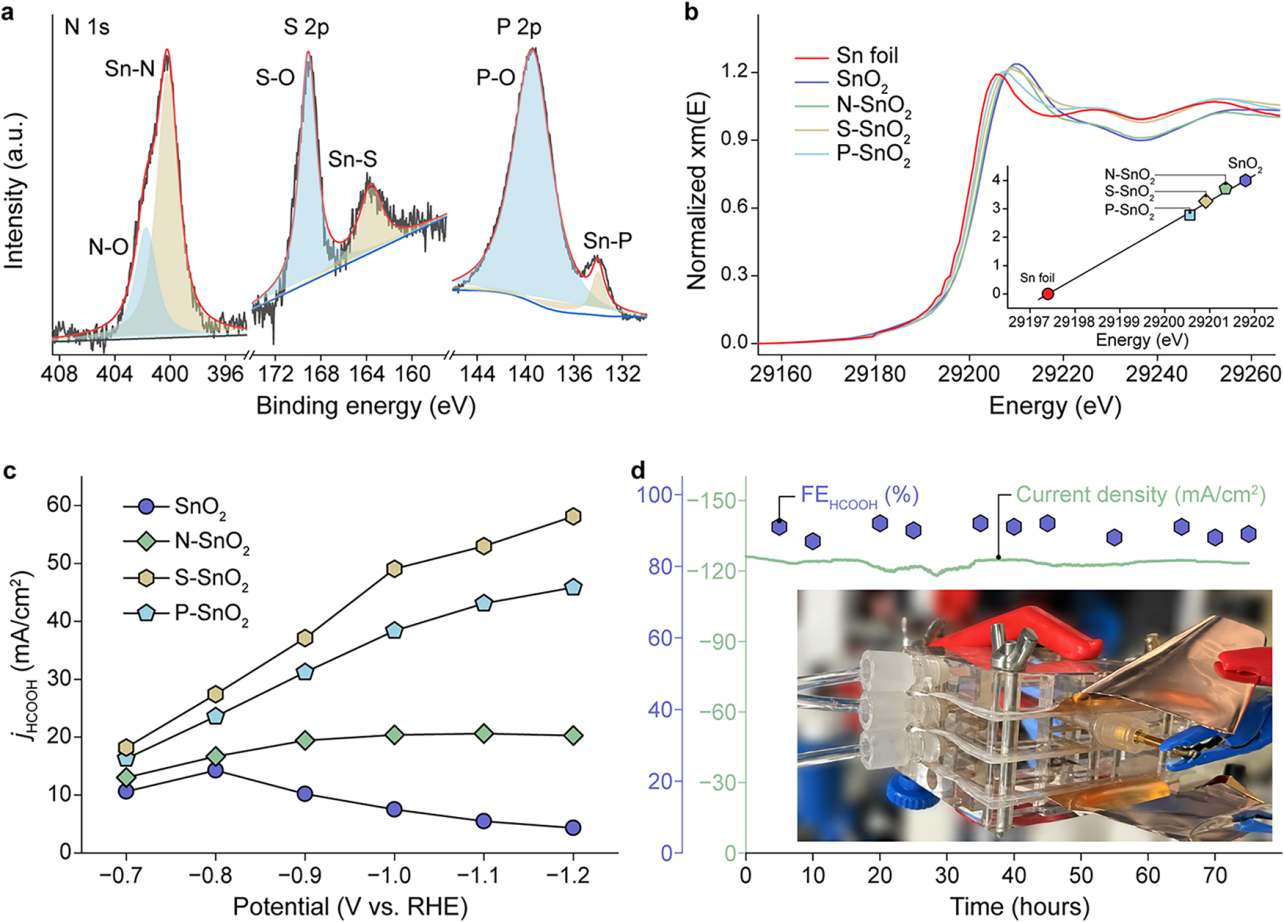
Structural and electronic modulation of SnO_**2**_ by nonmetal dopants and their impact on CO_2_ electroreduction. (a) High-resolution XPS spectra confirming the formation of Sn–N, Sn–S, and Sn–P bonds in N-, S-, and P-doped SnO_2_, respectively, along with signals from oxidized species (e.g., N–O, S–O, P–O). (b) Sn L_3_-edge XANES spectra showing a progressive redshift and decreased white line intensity from pristine to P-doped SnO_2_, indicative of reduced Sn oxidation states and modified electronic environments induced by heteroatom coordination. (c) Partial current densities for HCOOH production as a function of applied potential, highlighting enhanced CO_2_RR performance for doped catalysts, particularly S-SnO_2_. (d) Stability test of S-SnO_2_ in a flow-cell electrolyzer at −1.0 V vs RHE, revealing stable formate Faradaic efficiency (FE_HCOOH_, >90%) and constant current density over 50 h of continuous operation, insect shows the photograph of the flow-cell setup used in the electrolysis experiments.

To evaluate electrocatalytic performance under practical conditions, all tests were conducted in a three-compartment flow-cell reactor with continuous CO_2_ feed and efficient gas–liquid interface (Supporting Methods Section for setup details). The Faradaic efficiencies (FEs) for HCOOH formation were measured across a broad potential range (−0.7 to −1.2 V vs RHE). As shown in Figure S6, S-SnO_2_ exhibits the highest formate selectivity, maintaining FE_HCOOH_ > 90% over the full potential window. In contrast, P-SnO_2_ shows only moderate selectivity (∼70–80%), while N-SnO_2_ and SnO_2_ suffer from pronounced selectivity loss at more negative potentials due to increased hydrogen evolution and minor CO/CH_4_ formation. The corresponding partial current densities for HCOOH (*j*_HCOOH_) follow the trend: SnO_2_ < N-SnO_2_ < P-SnO_2_ < S-SnO_2_ (Figure 2c). Control experiments varying N, S, and P doping concentration (Figures S7–S10) show that while the formate activity dependence on concentration is nonmonotonic (e.g., for P), S remains the most effective across all tested ranges, underscoring a dopant-species-specific programming of interfacial water. Specifically, S-SnO_2_ reaches 60 mA cm^–2^ at −1.2 V vs RHE, representing more than a 4-fold increase over SnO_2_, confirming substantial enhancement in intrinsic activity. Under continuous operation, S-SnO_2_ also demonstrates remarkable stability, maintaining both FE_HCOOH_ > 90% and a stable current density of ∼130 mA cm^–2^ for 80 h in the flow cell without observable degradation (Figure 2d), underscoring its robustness under operationally relevant conditions. Notably, although P-SnO2 shows the strongest electronic perturbation/oxidation-state shift (Figure 2b), it does not deliver the highest formate activity (Figure 2c), indicating that electronic descriptors alone do not fully account for the observed trend and motivating additional analysis of interfacial hydration/proton delivery. To compare bulk transport/area effects, we performed EIS and Cdl-based ECSA analyses (Figures S11–S13). Doping markedly reduces the impedance relative to pristine SnO_2_, as expected; however, S-SnO_2_ and P-SnO_2_ exhibit similarly low impedances and comparable phase-angle responses across potentials, suggesting similar charge-transfer/transport characteristics. In parallel, Cdl values are comparable (2.73, 1.79, 3.36, and 3.65 mF cm^–2^ for SnO_2_, N-SnO_2_, S-SnO_2_, and P-SnO_2_, respectively), and P-SnO_2_ has the largest Cdl yet is not the best CO_2_RR catalyst. Together, these controls indicate that the S-specific enhancement cannot be attributed solely to conductivity or surface area, motivating the mechanistic analysis of interfacial hydration/proton-delivery regulation below. To elucidate the structural origin of the activity trend, we next examined the electronic and lattice-level response of Sn centers under operating conditions.

To elucidate the structural origin of the activity trend, we next examined the electronic and lattice-level response of Sn centers under operating conditions. *In situ* Raman spectroscopy (Figure S14) reveals three dominant vibrational modes corresponding to Sn–O lattice phonons: the E_g_ (∼476 cm^–1^), A_1g_ (∼631 cm^–1^), and B_2g_ (∼774 cm^–1^) modes.^29^ These phonons show distinct potential-dependent attenuation across the different samples. Pristine SnO_2_ and N-SnO_2_ exhibit significant peak broadening and intensity loss under cathodic bias, indicative of Sn–O bond disruption and progressive reduction. In contrast, S- and P-doped SnO_2_ retain sharper and more intense features, and enhanced resistance of the Sn–O framework against reductive degradation. These trends are corroborated by *operando* Sn L_3_-edge XANES measurements (Figure S15a), which tracks the dynamic oxidation-state changes of Sn centers. Upon applying −0.7 V vs RHE, all samples exhibit a reduction in white-line intensity, consistent with partial Sn^4+^ reduction. However, the magnitude of this shift (Δ*E*, Figure S15b) varies significantly: pristine SnO_2_ shows the largest change (0.26 eV), while P- and S- SnO_2_ exhibit much smaller shifts (0.08 and 0.10 eV, respectively). Taken together, the Raman and XANES results suggest that P and S dopants not only enhance the electronic delocalization and charge buffering capacity of the lattice but also stabilize the states of Sn active centers under CO_2_RR conditions.

To further explore the regulatory mechanism of N, S, and P doping on the CO_2_RR performance of SnO_2_ surface, we conducted operando ATR-SEIRAS measurements to monitor the evolution of CO_2_RR intermediate under progressively increasing cathodic potentials. (Figure 3a–d). All samples displayed vibrational features corresponding to *CO (∼1900 cm^–1^) and *COOH/*OCHO (∼1400 cm^–1^), confirming the formation of key intermediates for CO_2_-to-formate conversion.^30,31^ The CO-related band near ∼1900 cm^–1^ is relatively weak for some catalysts under our conditions; we therefore treat it as a CO-adsorption signature whose apparent intensity can vary with surface coverage and interfacial selection rules, rather than as a uniformly strong feature across all samples. Given that CO remains a minor product under our conditions, this CO-related/bridge-type adsorption feature on S-SnO_2_ is interpreted as a spectroscopic marker of a minor CO adsorption state rather than a necessary precursor in the dominant CO_2_-to-formate pathway. Notably, P-SnO_2_ exhibited the strongest and most persistent signals for both features. Because SEIRAS enhancement factors can vary between different Au films, these apparent intensity differences are interpreted qualitatively to monitor the formation and potential-dependent evolution of adsorbed intermediates, rather than as a direct quantitative comparison of surface coverage across catalysts. Nevertheless, the apparent persistence of intermediate-related features on P-SnO_2_ does not correlate with the highest catalytic activity, as P-SnO_2_ delivered only moderate formate production. In contrast, S-SnO_2_ achieved the highest formate selectivity and current density, although its intermediate-related SEIRAS response was not the most pronounced under our measurement conditions. Intriguingly, S-SnO_2_ also showed a unique response in the H_2_O bending vibration region (∼1650 cm^–1^). As the potential became more negative, the intensity of this H_2_O band increased markedly and then decreased, forming a distinct bell-shaped trend absent in the other samples. This observation indicates a pronounced potential-dependent restructuring of the interfacial hydration layer on S-SnO_2_, which is discussed in conjunction with PCET-related interfacial descriptors in the subsequent section.

**Figure 3:**
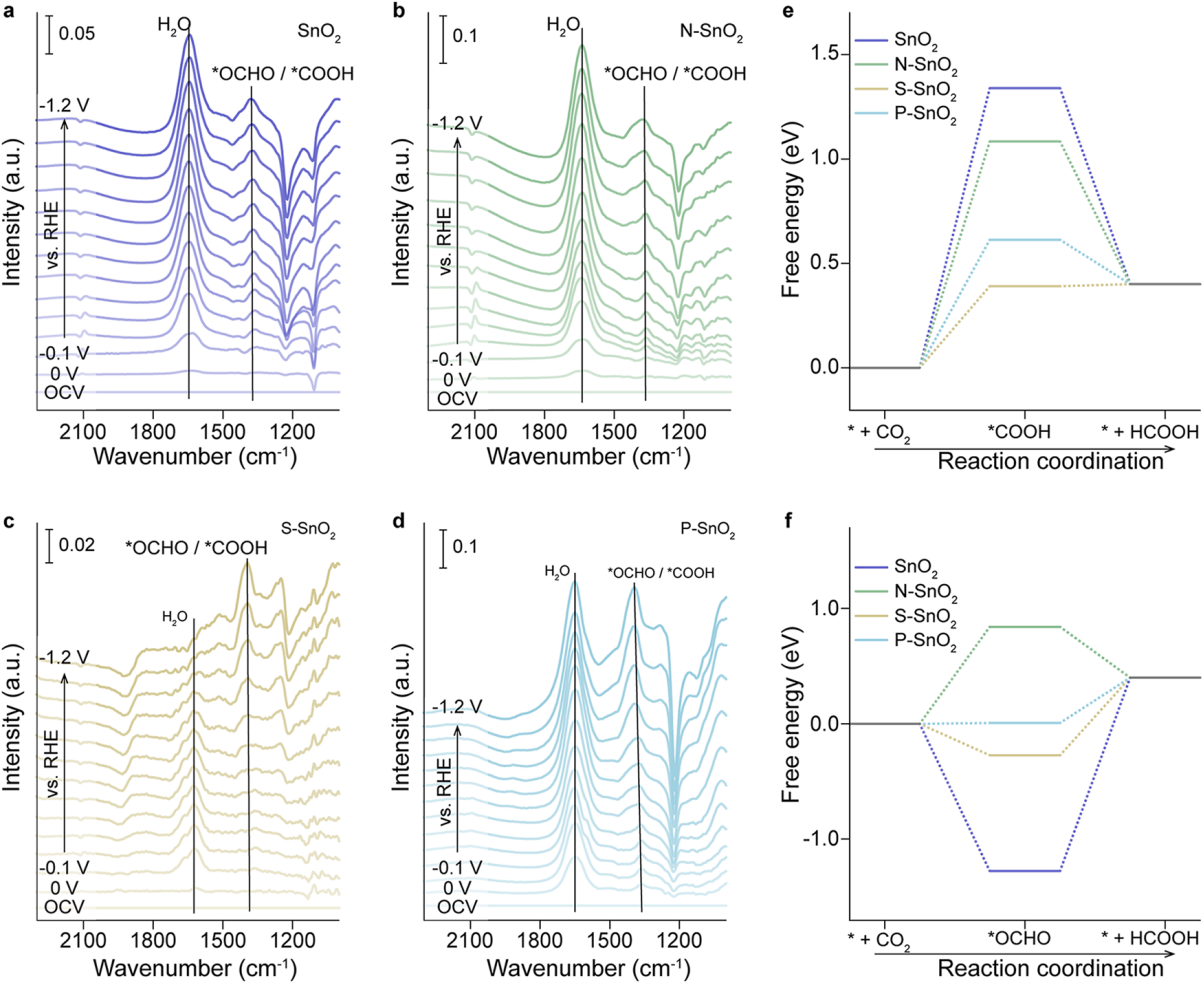
Interfacial intermediate evolution and theoretical reaction pathways on SnO_2_-based catalysts. (a–d) *Operando* ATR-SEIRAS spectra collected under increasing cathodic potentials for SnO_2_ (a), N-SnO_2_ (b), S-SnO_2_ (c), and P-SnO_2_ (d), showing potential-dependent evolution of vibrational bands assigned to a CO-related band (∼1900 cm^–1^), *OCHO/*COOH (∼1400 cm^–1^) and H_2_O (∼1650 cm^–1^). Electrolyte: CO_2_-saturated 0.5 M KHCO_3_. Spectra were collected from OCP to −1.2 V vs RHE with a 0.1 V interval, as indicated on the panels. (e) Free energy profile for HCOOH production via *COOH. (f) Alternative HCOOH pathway via *OCHO formation.

To further rationalize the discrepancy between intermediate binding behavior and catalytic performance, we performed DFT calculations to evaluate the thermodynamics of key CO_2_RR pathways (Figures 3e,f, and S16–S19). There are two reaction pathways for HCOOH production. Figures 3e,3f, respectively, show the energies of *COOH and *OCHO. Compared with the *COOH pathway, the *OCHO pathway on the surface of SnO_2_ and doped SnO_2_ is more favorable in terms of energy, which is consistent with the research results of Sn-based catalysts in the literature.^32–38^ It can be observed that for the path of *OCHO (Figure 3f), over P-SnO_2_ and S-SnO_2_, the potential-limiting step (PDS) is the second electron transfer process (*OCHO + H^+^ + e^–^), with energies of 0.32 and 0.67 eV, respectively. The surface of P-SnO_2_ has the lowest reaction free energy, which is inconsistent with the performance relationship shown in Figure 2c. To complement the formate pathways, we additionally evaluated the competing CO_2_-to-CO route via COOH/CO (Figure S20). The calculated energetics do not support a dominant CO-producing channel under our conditions, consistent with the minor CO product fraction and supporting that the CO-related band is not associated with a main reaction pathway. Furthermore, we analyzed the electronic and adsorption structures of the doped surfaces. PDOS calculations show that heteroatom doping introduces new states near the Fermi level, increasing surface reactivity. Bader charge and differential charge density analyses reveal that S-SnO_2_ induces more pronounced electron redistribution around Sn centers, consistent with enhanced local polarity. (Figures S21–S24) However, the calculated adsorption energetics and the apparent persistence of intermediate-related SEIRAS features on P-SnO_2_ do not translate into the highest activity, reinforcing the notion that static adsorption strength or apparent intermediate-related SEIRAS intensity alone is an insufficient descriptor for catalytic efficiency. Taken together, these experimental and theoretical findings support a cooperative picture in which heteroatom doping modulates both the surface electronic structure (and thus adsorption energetics/local fields) and the interfacial hydration environment; however, our operando and kinetic results indicate that hydration/proton-delivery descriptors provide the missing dimension required alongside electronic descriptors to reconcile the adsorption-reactivity inconsistency and to account for the exceptional formate performance of S-SnO_2_. Specifically, dopant-induced restructuring of the H_2_O layer and its impact on proton-coupled electron transfer (PCET) emerge as key determinants of CO_2_RR selectivity and kinetics. This contradiction underscores the necessity of adopting a holistic approach to establish a precise quantitative structure–activity relationship in CO_2_RR. Specifically, beyond comprehensively elucidating the intermediate adsorption behavior and electron transfer pathways, both governed by the catalyst’s surface electronic structure, it is equally critical to systematically investigate how the catalyst’s surface electronic structure modulates the electrochemical interface microenvironment, particularly its influence on the PCET step.

To further elucidate how interfacial H_2_O governs CO_2_RR kinetics and selectivity, we analyzed the O–H stretching region (3080–3640 cm^–1^) of *operando* ATR-SEIRAS spectra (Figures 4a,b and S25).^39,40^ Rather than treating “ordered” and “disordered” H_2_O as a purely qualitative binary assignment, we further define these populations according to their hydrogen-bonding environments within the ν_OH_ envelope. Specifically, the lower-frequency strongly H-bonded component and the medium-frequency moderately H-bonded component are grouped as ordered H_2_O, reflecting a more connected hydrogen-bond network, whereas the higher-frequency weakly H-bonded/nearly non-hydrogen-bonded component is assigned to disordered H_2_O, reflecting weakened hydrogen bonding and increased local structural freedom under interfacial perturbation.^41^ Quantifying the disordered H_2_O population reveals that cathodic bias increases the fraction of H-bond-disrupted interfacial H_2_O for all samples, while the magnitude of this transition is substantially larger on S-SnO_2_ (Figure 4c), indicating a more connectivity-disrupted hydration environment under operating bias. To connect the spectral evolution to atomic-scale interfacial structure, we performed AIMD simulations. The vibrational density of states (Figure 4d), shows that S-SnO_2_ exhibits intensified high-frequency O–H vibrations, consistent with strengthened H_2_O–surface interactions and a perturbed hydrogen-bond network.^16,42^ Complementary radial distribution functions (RDFs), further reveal broadened first-shell features and strongly damped medium-range oscillations in both Sn–H and Sn–O correlations on S-SnO_2_, consistent with a connectivity-disrupted interfacial solvation shell relative to SnO_2_, N-SnO_2_, and P-SnO_2_ (Figure 4e,f).^43–45^ Importantly, we do not infer molecular orientation solely from ν_OH_ deconvolution because ATR-SEIRAS intensities are governed by the surface-selection rule and depend strongly on dipole orientation (approximately cos^2^ θ).^46^ Instead, the H-down-rich orientational bias on S-SnO_2_ is supported independently by AIMD-derived two-dimensional angular distribution maps (cos θ_1_ – cos θ_2_, Figures 4g and S26), which quantify an asymmetric configuration with one O–H preferentially aligned toward the surface while the other is directed away. Together, the hydration-shell connectivity disruption (RDF/VDOS) and orientational bias (cos θ maps) identify the S-SnO_2_ interface as connectivity-disrupted but orientation-biased (H-down-rich), providing a structural basis for enhanced directional proton delivery and accelerated PCET. The energetic implications of this solvent reorganization were further probed by DFT calculations. Among all samples, S-SnO_2_ exhibits the most exergonic H_2_O adsorption energy (−0.97 eV, Figure S27) and a shortened S···H–O hydrogen bond distance of 1.91 Å (Figure S28), forming a compact prereactive geometry conducive to H_2_O activation. Most notably, the calculated H_2_O dissociation barrier on S-SnO_2_ is nearly negligible (0.009 eV, Figure S29), orders of magnitude lower than those of N-SnO_2_ (1.11 eV) and pristine SnO_2_ (1.32 eV). This nearly barrierless dissociation process highlights the kinetic advantage of the S-induced interfacial environment in promoting surface hydroxyl generation and mobile proton supply during CO_2_RR.

**Figure 4:**
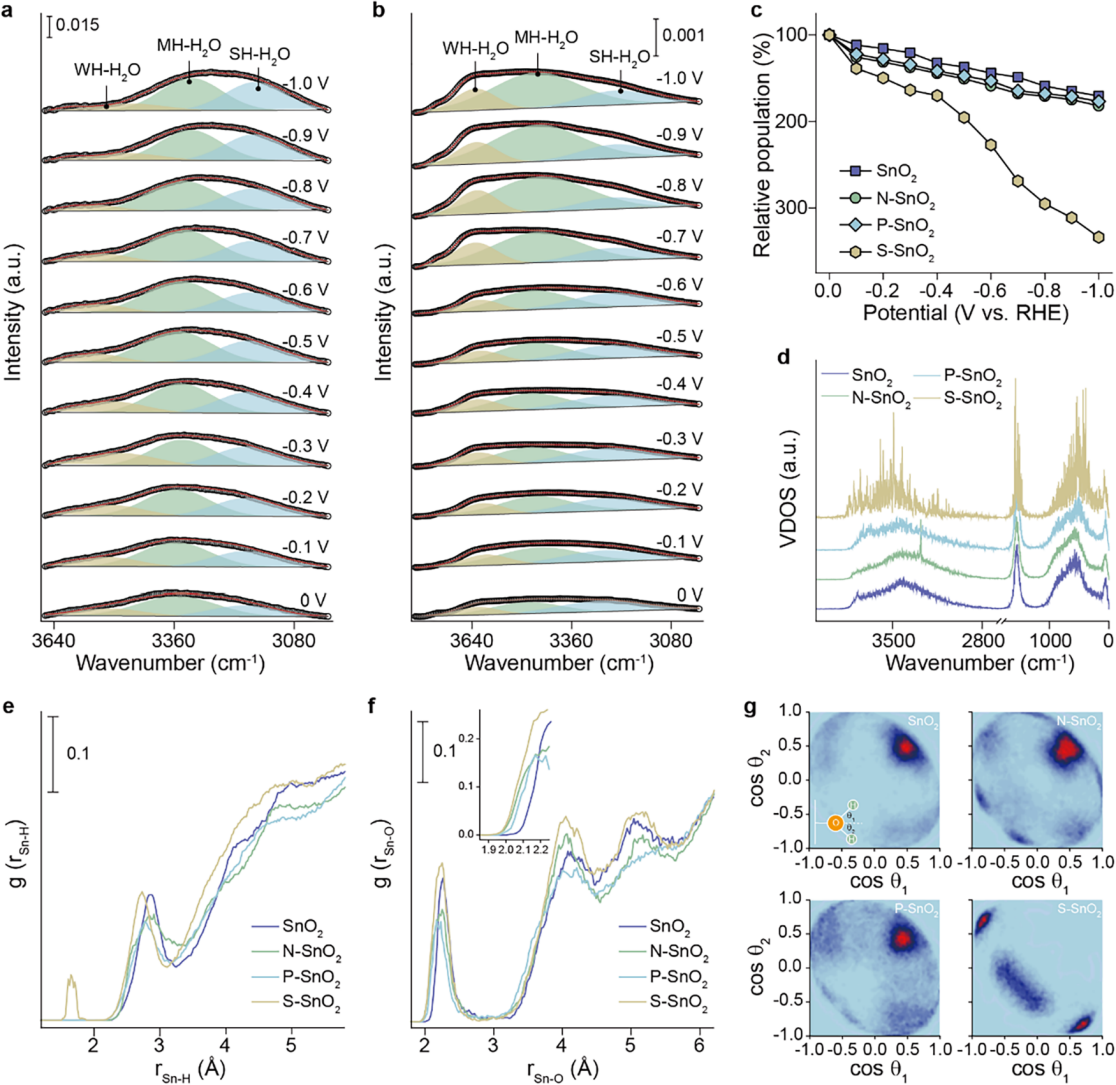
Dopant-mediated restructuring of the interfacial H_2_O network. (a, b) *Operando* ATR-SEIRAS spectra of the O–H stretching region (3080–3640 cm^–1^) recorded under increasing cathodic potentials for P-SnO_2_ (a) and S-SnO_2_ (b). The ν_OH_ envelopes (3080–3640 cm^–1^) are deconvoluted into contributions associated with strongly hydrogen-bonded interfacial H_2_O, WH-H_2_O, MH-H_2_O, and SH-H_2_O denote weakly H-bonded, moderately H-bonded, and strongly H-bonded interfacial H_2_O populations, respectively. The ν_OH_ envelopes are deconvoluted into contributions associated with strongly H-bonded H_2_O, weakly H-bonded/H-bond-disrupted H_2_O, and a high-frequency free–OH-like shoulder (fitting constraints and component ranges are provided in Figure S25). (c) Quantified evolution of disordered H_2_O populations relative to open-circuit potential (OCP) across the four samples. (d) Vibrational density of states (VDOS) from AIMD simulations, where S-SnO_2_ exhibits intensified high-frequency O–H vibrations, consistent with stronger H_2_O-surface interactions. (e, f) Radial distribution functions (RDFs) for Sn–H and Sn–O, showing shorter, more disordered H_2_O adsorption on S-SnO_2_. (g) Two-dimensional angular distribution maps of H_2_O dipoles projected onto cos θ_1_ and cos θ_2_ for each catalyst surface. The H-down-rich orientational bias is assessed independently from AIMD angular distribution statistics (panel (g) and Figure S26), consistent with the ATR-SEIRAS surface-selection rule (intensity ∝ cos^2^ θ).

To directly connect dopant-regulated interfacial H_2_O structures with CO_2_ reduction kinetics, we evaluated CO_2_RR performance and proton-transfer sensitivity for all four catalysts (Figure 5). Under CO_2_-saturated conditions (Figure 5a), S-SnO_2_ delivers the largest cathodic current density together with the smallest Tafel slope (155.7 mV dec^–1^, Figure 5b), indicating a distinct kinetic regime in which the barrier for the rate-determining step is substantially lowered. The low slope is consistent with a fast initial electron-transfer step followed by a chemical protonation of *OCHO/*COOH intermediates. H/D isotope substitution further reveals that SnO_2_, N-SnO_2_ and P-SnO_2_ exhibit pronounced decreases in CO_2_RR current density as the D_2_O fraction increases (Figure 5c), evidencing a strong kinetic isotope effect and highlighting their reliance on bulk proton supply. In contrast, S-SnO_2_ shows only a modest, gradual decline in current upon D_2_O addition, implying that proton delivery is buffered by the preorganized H-down interfacial H_2_O network rather than limited by proton diffusion from the bulk. This interfacial robustness is also reflected in the product distribution: at −0.8 V vs RHE, the formate Faradaic efficiencies of SnO_2_, N-SnO_2_ and P-SnO_2_ decrease noticeably with increasing D_2_O content, whereas S-SnO_2_ maintains both the highest FE_HCOOH_ and the smallest isotope-induced variation (Figure 5d). Together with the structural and dynamical descriptors of the S-modified H_2_O layer, these electrochemical trends support a scenario in which S-SnO_2_ sustains CO_2_RR through a solvent-organized, interface-confined proton-transfer pathway that efficiently feeds PCET to CO_2_-derived intermediates.

**Figure 5:**
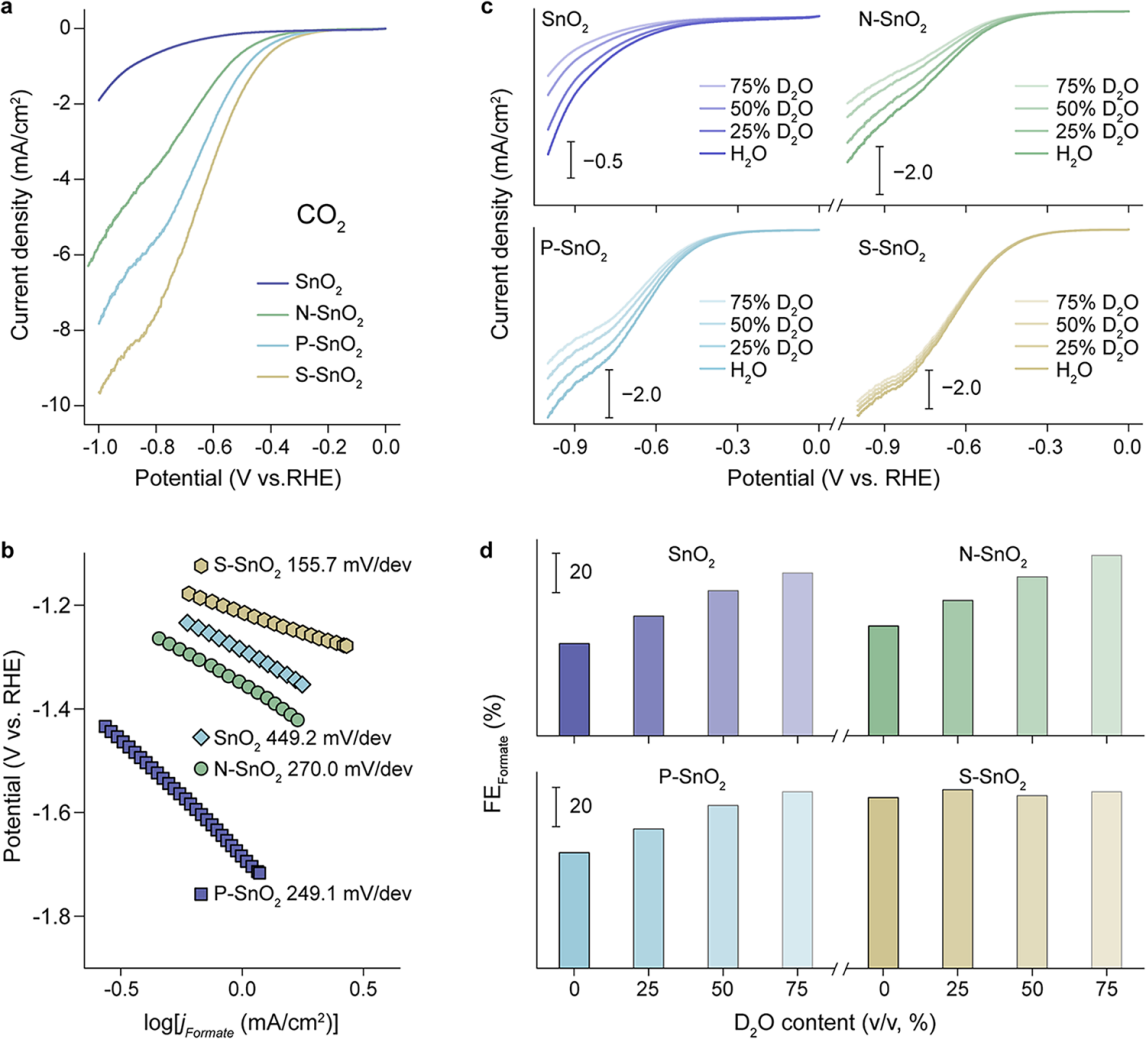
Electrochemical kinetics of HER and CO_2_RR reveal dopant-regulated proton transfer mechanisms. (a–d) CO_2_ reduction reaction (CO_2_RR) performance in CO_2_-saturated electrolyte. (a) LSV curves. (b) Corresponding Tafel plots. (c) KIE analysis under CO_2_RR conditions. (d) Faradaic efficiencies for formate at −0.8 V versus RHE as a function of D_2_O fraction.

To verify that this dopant effect is selective for CO_2_RR rather than a general acceleration of proton-involving reactions, we also evaluated HER in Ar-saturated electrolyte (Figures S30–S31).^47,48^ In this case, S-SnO_2_ exhibits the lowest HER current density despite its proton-rich interface, and the isotope effect follows the opposite trend: HER activity on SnO_2_, N-SnO_2_ and P-SnO_2_ is more strongly suppressed in D_2_O than on S-SnO_2_. These control experiments corroborate that S-doping does not simply increase proton availability but reshapes the electrochemical double layer so that the reorganized interfacial H_2_O specifically favors the PCET pathway for CO_2_-to-formate conversion, while rendering the sequence of steps required for H–H bond formation kinetically unfavorable.

## Conclusion

In this study, we establish a solvation-structure-directed paradigm for CO_2_ electroreduction by systematically engineering the interfacial environment of SnO_2_ catalysts through nonmetal heteroatom doping. Integrating *operando* spectroscopy, ab initio molecular dynamics simulations, and electrochemical kinetics, we demonstrate that sulfur doping uniquely promotes disordered yet H-down-oriented H_2_O configurations at the catalyst-electrolyte interface. This restructured hydration layer lowers the barrier for interfacial H_2_O dissociation and enables efficient proton-coupled electron transfer (PCET), thereby overriding conventional activity trends based on intermediate adsorption energies or surface oxidation states. Notably, although P-SnO_2_ exhibits the strongest electronic perturbation and intermediate binding affinity, it fails to deliver superior catalytic performance, highlighting the decisive role of interfacial H_2_O dynamics in governing reaction pathways. Our findings underscore that solvation structure, its orientation, disorder, and reactivity, can serve as a tunable vector to modulate electrochemical selectivity and kinetics. This insight opens a new direction for electrocatalyst design, where dynamic solvent–catalyst interactions are actively harnessed alongside conventional surface engineering.

## Supplementary Material



## Data Availability

All experimental and spectroscopic data are included in Supporting Information. Source data are provided with this paper.

## Notes

Schematic illustration of the fabrication procedures for heteroatom-doped S-SnO_2_ catalysts; powder X-ray diffraction patterns of SnO2 and heteroatom-doped samples; elemental mapping of pristine and heteroatom-doped SnO2 catalysts; kinetic isotope effect (KIE) analysis using H_2_O/D_2_O electrolytes (PDF)
