# Supplementary material for: Heteroatom Doping Restructures Interfacial H2O to Resolve Mechanistic Contradictions in CO2 Electroreduction
Source: J Am Chem Soc. 2026 Jul 20;148(30):32335–43. doi: 10.1021/jacs.6c08293 (PMC13450404; doi:10.1021/jacs.6c08293)
Supplement: Supplementary file 1 [file ja6c08293_si_001.pdf]

Supplementary Information for

Heteroatom doping restructures interfacial H_2O to resolve mechanistic contradictions in CO_2 electroreduction

Lingyue Liu[1,#], Haihui Lan[2,#], Li Li[1], Weijue Wang[3,4], Yuhang Jin[5], Guoqin Liu[6], Yuhang Liu[5], Wenqiang Yang[6], Ming Zhao[5], Jie Ding[1], Hongbin Yang[5,*], Yanqiang Huang[3,4,*], Xinliang Feng[1,6,*]

[1]Max Planck Institute of Microstructure Physics, Halle (Saale) 06120, Germany.

[2]Department of Chemistry, Massachusetts Institute of Technology, Cambridge, MA, United States.

[3]State Key Laboratory of Catalysis, Dalian Institute of Chemical Physics, Chinese Academy of Sciences, Dalian 116023, China.

[4]University of Chinese Academy of Sciences; Beijing 100049, China.

[5]School of Materials Science and Engineering, Suzhou University of Science and Technology, Suzhou 215009, China.

[6]Faculty of Chemistry and Food Chemistry & Center for Advancing Electronics Dresden (cfaed), Technische Universität Dresden, Dresden 01062, Germany.

[#]These authors contributed equally.

*Corresponding author. yanghb@mail.usts.edu.cn (H. Yang), yqhuang@dicp.ac.cn (Y. Huang) and Xinliang.feng@mpi-halle.mpg.de (X. Feng)

Method

Chemicals. Tin chloride ($SnCl_4$), Urea, Sodium DL-lactate, ethanol, thiourea, sodium hypophosphite, potassium bicarbonate ($KHCO_3$), and NafionTM 115 solution (5 wt.% in lower aliphatic alcohols and water) were purchased from Sigma Aldrich and used directly without further purification.

Synthesis of SnO_2, N-SnO_2, P-SnO_2 and S-SnO_2. In a typical synthesis, 40 g of ice and 3.91 g (15 mmol) of Tin chloride ($SnCl_4$) were heat until dissolved, then 5 g urea were added into the solution under vigorous stirring. Afterwards, the mixture was added to the sodium lactate 5 ml. The obtained transparent solution was transferred to a Teflon-lined stainless steel autoclave to perform solvothermal process at 200 °C for 20 h. After cooling down to room temperature naturally, the solid product was collected by centrifugation and washed with deionized water and ethanol for three times. The sample was finally dried in a vacuum oven for 12 h at 80 °C.

Take a certain amount of the above powder and expose it separately in a tube furnace to the vapor precursors of urea (N source), thiourea (S source), and sodium hypophosphite (P source). The thermally treated temperature on the doped side is set at 260 ^{o}C, while the temperature on the SnO_2 side is set at 200 ^{o}C. Keep it at this temperature for 2 h to prepare N-SnO_2, P-SnO_2, and S-SnO_2.

Characterizations. The X-ray diffraction (XRD) patterns were recorded on a Bruker D8 diffractometer in the 2θ range of 10 to 80°, employing Cu Kα radiation with a wavelength of 1.5406 Å. The morphological information was collected by transmission electron microscopy (TEM, JEOL JEM-2100F) equipped with energy-dispersive X-ray spectroscopy (TEM-EDS, JEOL JEM-2100F). The chemical states of the samples were probed by X-ray photoelectron spectroscopy (XPS) on an ESCALAB 250 photoelectron spectrometer (Thermo Fisher Scientific), utilizing a monochromatic Al Kα X-ray beam with an energy of 1486.6 eV. All binding energies were referenced to the C 1s peak at 284.6 eV. X-ray absorption spectroscopy (XAS) was collected by employing synchrotron radiation light source at BL14W1 beamline of Shanghai Synchrotron Radiation Facility (SSRF) at room temperature. Energy calibration was

performed with a Sn foil standard by shifting all spectra to a glitch in the incident intensity. Fluorescence spectra were recorded using a seven-element Ge solid state detector. The XAFS data were recorded in fluorescence excitation mode using a Lytle detector, and the spectra of all references were collected in the transmission mode. The acquired XAFS data were analyzed by Athena and Artemis software, according to the standard procedures.

Electrochemical CO_2RR in H-cell. Electrochemical CO_2 reduction measurements were conducted in a three-compartment flow-cell reactor using a CHI 760e electrochemical workstation. The as-prepared catalyst-coated carbon paper (geometric area: 1 cm × 1 cm) served as the working electrode, a platinum plate as the counter electrode, and an Ag/AgCl electrode as the reference electrode. All potentials were converted to the reversible hydrogen electrode (RHE) scale. The catholyte and anolyte were separated by an ion-exchange membrane, and 0.5 M $KHCO_3$ aqueous solution was continuously circulated through the cathode chamber. Prior to electrolysis, the catholyte was saturated with CO_2 by bubbling at 20 mL min^{-1} for 30 min. During operation, CO_2 gas was continuously supplied to the cathode chamber to maintain a steady-state gas-liquid interface and ensure sufficient mass transport. Gaseous products were continuously carried by the outlet gas stream and analyzed online using a gas chromatograph equipped with appropriate detectors. Liquid products were quantified after electrolysis using ^1H nuclear magnetic resonance (^1H NMR) spectroscopy (Bruker Avance NEO 400 MHz), with dimethyl sulfoxide (DMSO) as the internal standard.

CO_2 reduction reaction electrochemical measurements. Electrochemical measurements were carried out at ambient temperature and pressure on a rotating disc electrode system (Pine Inc.) using a CHI 760e potentiostat. A three-electrode cell configuration was employed with a working electrode of glassy carbon rotating disc electrode (RDE) of 5 mm diameter, a counter electrode of graphite rod (5 mm diameter), and a Ag/AgCl reference electrode. To prepare the catalyst ink, 5 mg of catalyst and 25 μL of 5 wt.% Nafion™ solution were introduced into 975 μL of water-isopropanol solution with equal volume of water and isopropanol and sonicated for 3 h. An aliquot

of 8 μL of the catalyst ink was applied onto a glassy carbon RDE and allowed to dry naturally in air. A CO_2-saturated electrolyte was prepared by purging CO_2 (99.99%) into 0.5 M $KHCO_3$ aqueous solution for 30 min, and a flow (100 cc/min) of CO_2 was maintained over the electrolyte throughout the electrochemical measurements. All potentials here is relative to the reversible hydrogen electrode (RHE) scale unless stated otherwise. The linear sweep voltammograms (LSVs) were collected at a scan rate of 5 mV/s. Cyclic voltammetry (CV) curves were collected at a scan rate of 20 mV/s under CO_2-saturated 0.5 M $KHCO_3$ electrolyte. All current densities were normalized to the geometric area of the electrode.

***In-situ* ATR-SEIRAS measurements.** The in-situ ATR-SEIRAS experiments were performed using a customized electrochemical cell. Typically, 1 mg of the catalyst was dispersed in a mixture containing 1.9 mL DI-H_2O, 1.9 mL ethanol, and 0.2 mL of 5wt.% Nafion under ultrasonication for 1 hour to produce an ink with a catalyst concentration of 0.25 mg/mL. Subsequently, 100 μL of the catalyst ink was applied onto an Au film serving as the working electrode. An Ag/AgCl electrode (KCl-saturated) and a graphite rod were employed as the reference electrode and counter electrode respectively. The electrolyte was purified through an electrolysis process by maintaining at a constant negative current on an Au foil working electrode to remove any potential metal impurities. All spectroscopic measurements were acquired at a resolution of 4 cm−1 with at least 64 coadded scans using a Thermo Fisher Nicolet is20 FTIR spectrometer equipped with a liquid nitrogen cooled MCT detector. Electrochemical measurements were conducted using a CHI 760e equipment while impedance measurements were carried out at the beginning of each experiment, actively correcting for internal resistance (typically ~30 Ω) throughout all experiments. Reference spectra were obtained at CO_2 saturated solution (-0.6 V_{RHE}) in $KHCO_3$ (0.5 M). Unless otherwise stated, all reported potentials are referred to RHE.

***Operando* XAFS measurements.** The *operando* Sn L_3-edge absorption spectra were collected at different cathodic potentials on the BL 14W1 station at the Shanghai Synchrotron Radiation Facility (SSRF). Initially, a homogeneous catalyst ink was

prepared by ultrasonically mixing 5 mg of catalyst with 30 μL of nafion and 970 μL of ethanol. Subsequently, the catalyst ink was uniformly brushed onto one side of a carbon paper that was placed on a heating plate at 50 °C. The active area of the catalyst layer was 1 cm^2 with a mass loading of 3 mg/cm^2. It is important to note that the catalyst ink should not be deposited on the other side of the carbon paper to ensure proper contact between the catalyst and electrolyte solution. Next, the prepared working electrode was mounted onto an *operando* XAS fluorescence cell with a three-electrode configuration, using graphite rod and Ag/AgCl electrode (KCl-saturated) as counter and reference electrode, respectively. During *operando* XAS measurements, CO_2 was continuously bubbled at a flow rate of 20 sccm to saturate the 0.5 M $KHCO_3$ solution (50 mL). All *operando* XAS spectra were collected in the fluorescence mode at room temperature under constant potential (-0.6 V_{RHE}) for various reaction times.

***In-situ* Raman measurements.** *In-situ* Raman spectroscopy measurements were conducted in a custom-designed three-electrode SERS flow cell with a saturated Ag/AgCl electrode as the reference electrode and a graphite rod as the counter electrode in the anode chamber. During the measurements, the electrolyte was constantly purged with CO_2 gas and circulated across the cell using a peristaltic pump. The Raman measurements were performed on a LabRAM HR Evolution microscope (Horiba Jobin Yvon) with a 532 nm laser, a 50× objective, a monochromator (600 grooves/mm grating), and a CCD detector.

The First-principles Calculations. Density functional theory (DFT) calculations were executed using the Vienna Ab-initio Simulation Package (VASP).[1] The core-electron interactions were described by the projector augmented wave (PAW) method, and the Perdew-Burke-Ernzerhof (PBE) functional.[2] was applied for the exchange-correlation interactions. A plane wave energy cutoff of 450 eV was chosen.[3] The large-enough convergence criteria were set to 10^{-6} eV for electronic iterations and a force threshold of 0.01 eV/Å for ionic relaxations. The DFT-D3 dispersion correction was incorporated to account for van der Waals interactions.

For adsorption, we use the formula as following:

$$\Delta E_{ads} = \Delta E_{Total} - \Delta E_{slab} - \Delta E_{mol}$$

where E_{Total} represents the total energy of the total system complex, E_{slab} is the energy of the isolated surface, and E_{mol} is the energy of the molecule in vacuum.

$$\Delta G = \Delta E + \Delta E_{ZPE} - \mathrm{T}\Delta S + \Delta U_{0\rightarrow 300K}$$

where ΔE, ΔE_{ZPE}, ΔS, $\Delta U_{0\rightarrow 300\mathrm{K}}$, and T represent the calculated reaction energy, the zero-point energy correction, the entropy differences, the thermal correction to energy under 300 K without ZPE, and the temperature (300 K), respectively.

The simulated solid-liquid interface model consisted of a six-layer metal slab containing 96 Sn atoms and one specific heteroatom, interfaced with an explicit solvent box of 130 water molecules. The dimensions of the solvent region were carefully constructed to reproduce the standard experimental liquid water density of 1 g/cm^3. To realistically simulate the bulk properties of the substrate while allowing surface reactions, the bottom four atomic layers of the Sn slab were constrained in their bulk positions, whereas the top two Sn layers, the heteroatom, and all the water molecules were allowed to fully relax during the simulations.

All dynamic simulations were carried out in the canonical (NVT) ensemble. The system was initially heated to the target temperature utilizing a velocity-scaling method. Once the desired temperature was reached, a Nosé-Hoover thermostat was employed to strictly maintain the thermal equilibrium of the system. The equations of motion were integrated with a time step of 1 fs. A pre-equilibration run of 5 ps was first performed to allow the interfacial structure to relax and the thermodynamic properties to stabilize. Subsequently, a continuous production run of 10 ps was carried out to guarantee a comprehensive and statistically reliable sampling of the phase space for further analysis.

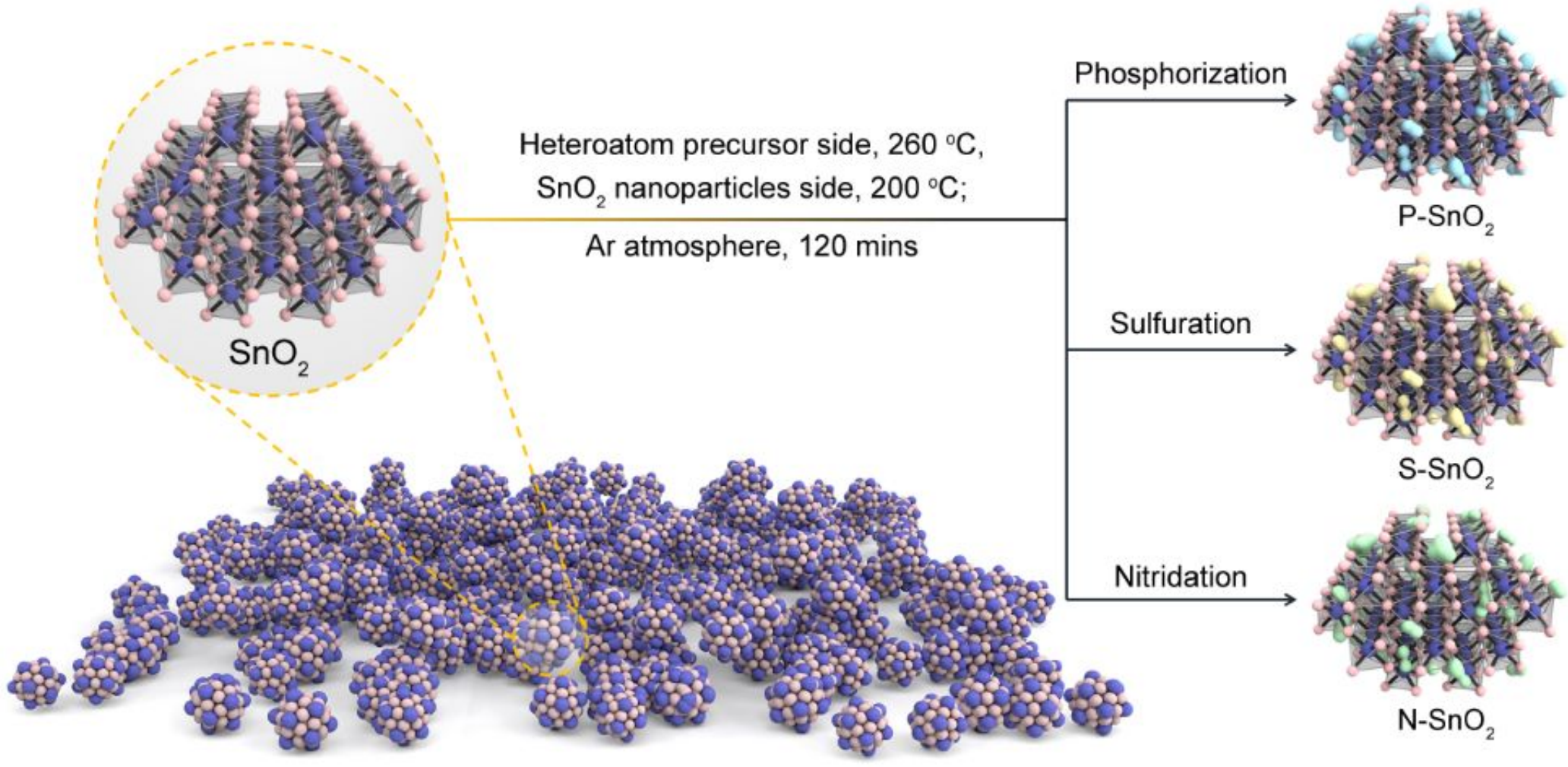

Supplementary Fig. 1 | Schematic illustration of the fabrication procedures for heteroatom-doped S-SnO_2 catalysts. SnO_2 nanoparticles were thermally treated with heteroatom precursors under an Ar atmosphere using a dual-zone configuration, where the precursor side was maintained at 260 °C and the SnO_2 side at 200 °C for 120 min. Depending on the precursor employed, phosphorization, sulfurization, or nitridation processes yielded P-SnO_2, S-SnO_2, and N-SnO_2, respectively.

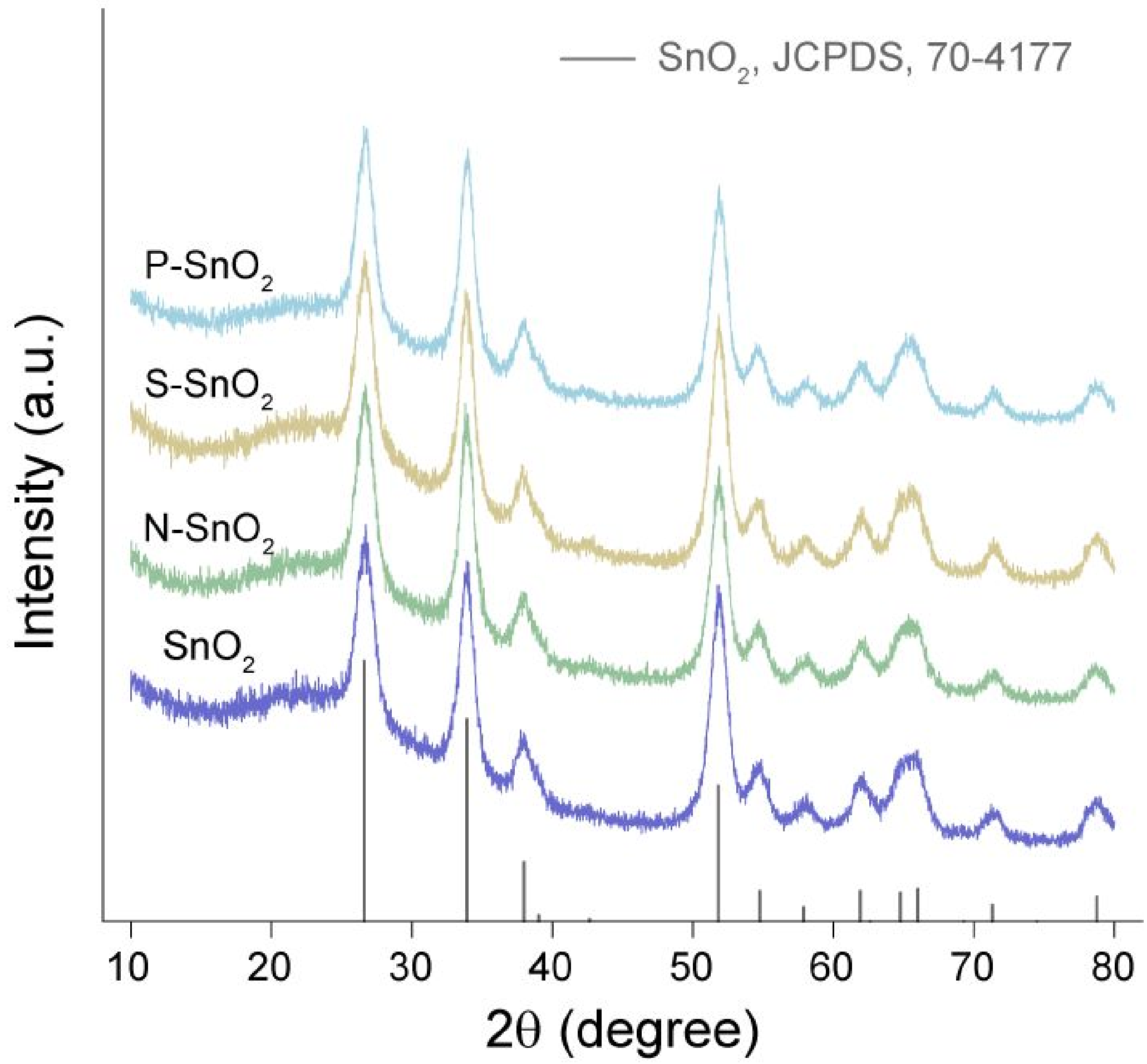

Supplementary Fig. 2 | Powder X-ray diffraction patterns of SnO_2 and heteroatom-doped samples. XRD patterns of pristine SnO_2 and N-, S-, and P-doped SnO_2 catalysts. All diffraction peaks can be indexed to tetragonal rutile SnO_2 (JCPDS No. 70-4177), with no detectable impurity phases observed after doping.

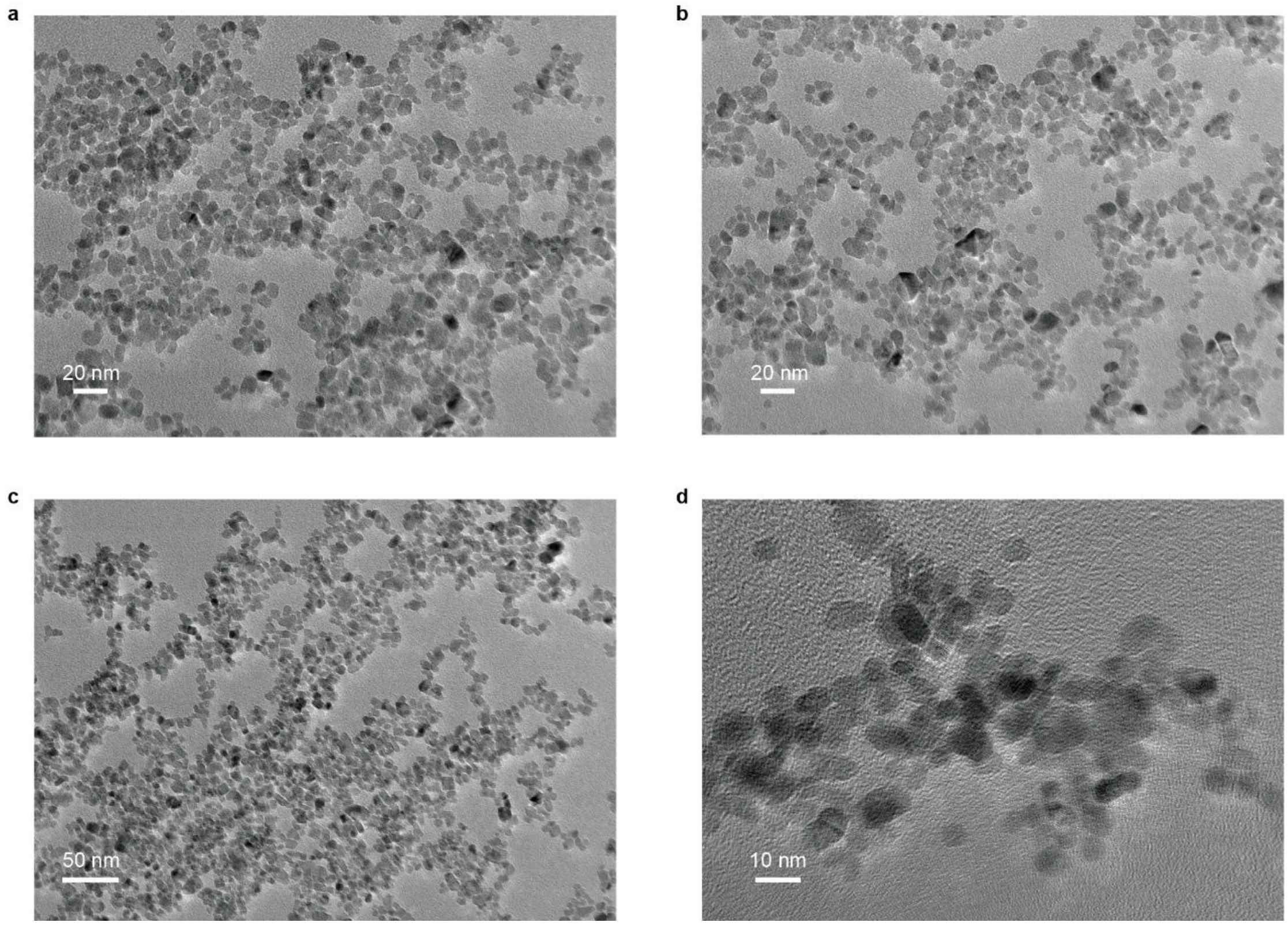

Supplementary Fig. 3 | Transmission electron microscopy (TEM) images of pristine and heteroatom-doped SnO_2 catalysts. TEM images of (a) SnO_2, (b) N-SnO_2, (c) S-SnO_2, and (d) P-SnO_2. All samples consist of uniformly dispersed nanoparticles with comparable sizes and no observable aggregation or phase segregation after heteroatom incorporation.

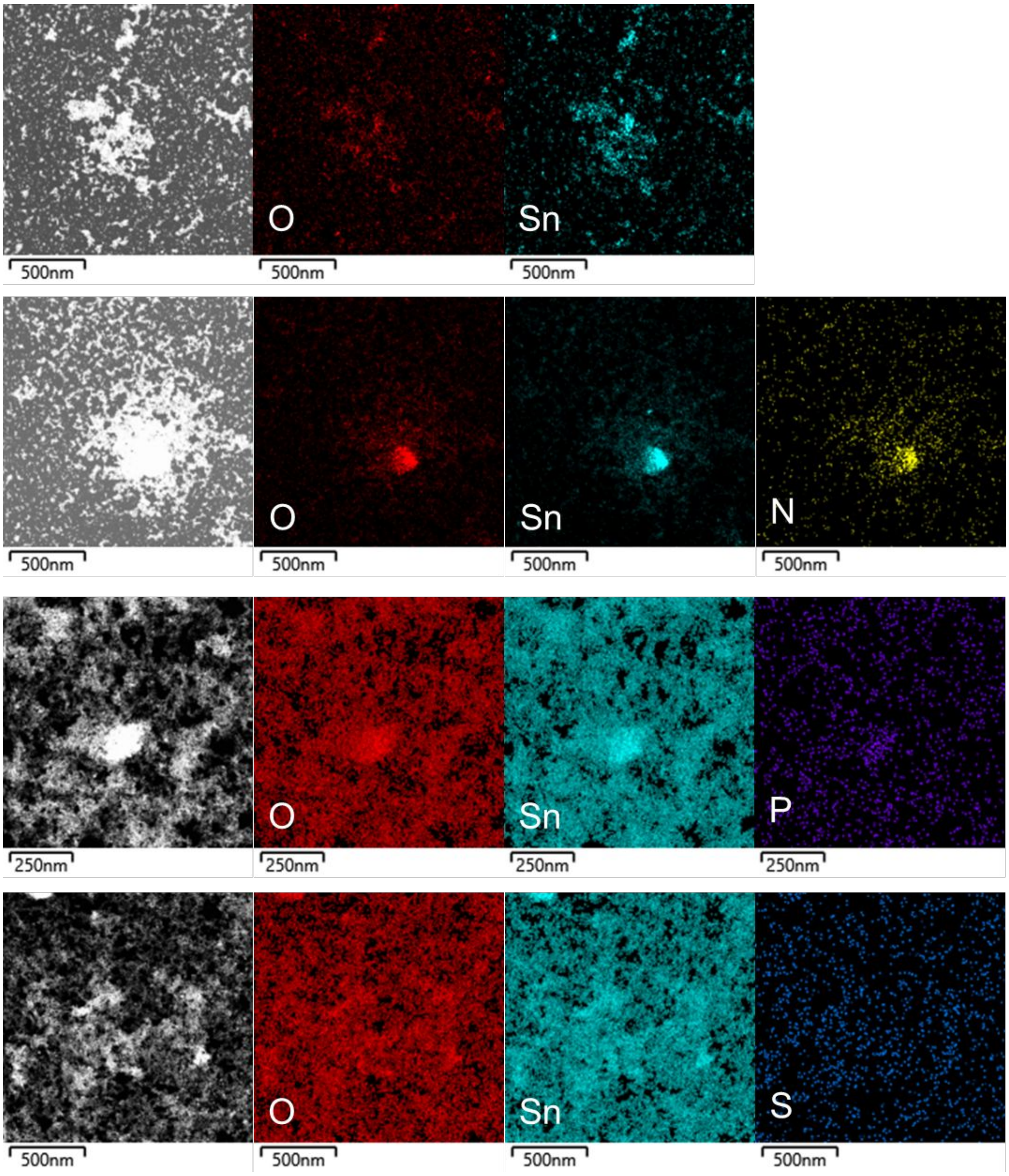

Supplementary Fig. 4 | Elemental mapping of pristine and heteroatom-doped SnO_2 catalysts. The O and Sn signals are uniformly distributed across all samples. For the doped catalysts, N, P, and S are homogeneously dispersed without detectable aggregation or phase segregation, indicating successful incorporation of heteroatoms into the SnO_2 framework rather than formation of separate impurity phases.

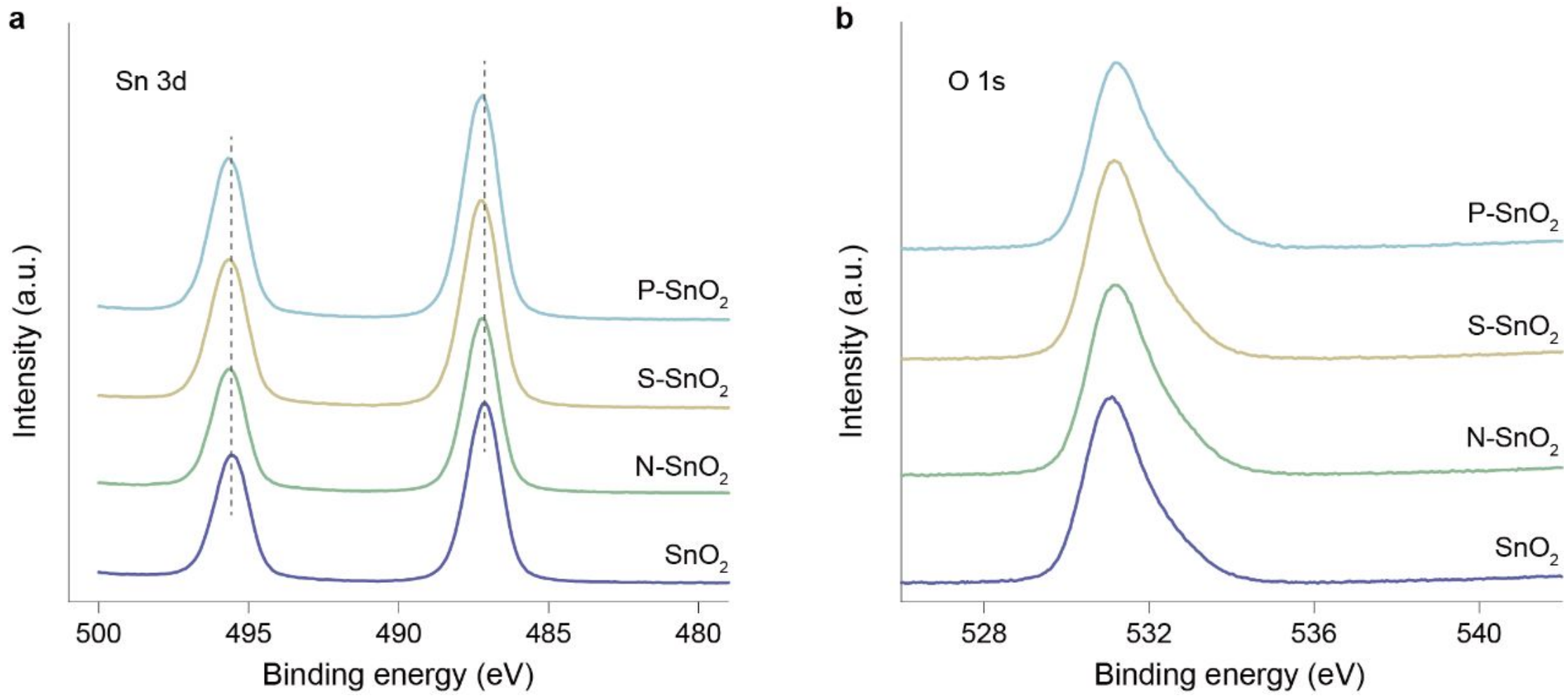

Supplementary Fig. 5 | High-resolution X-ray photoelectron spectroscopy (XPS) spectra of pristine and doped SnO_2 catalysts. Sn 3d core-level spectra (a) and O 1s spectra (b) of SnO_2, N-SnO_2, S-SnO_2, and P-SnO_2. The Sn 3d peaks exhibit slight shifts upon heteroatom incorporation, indicating modulation of the local electronic environment around Sn centers. Corresponding changes in the O 1s region further suggest altered Sn-O bonding characteristics after doping.

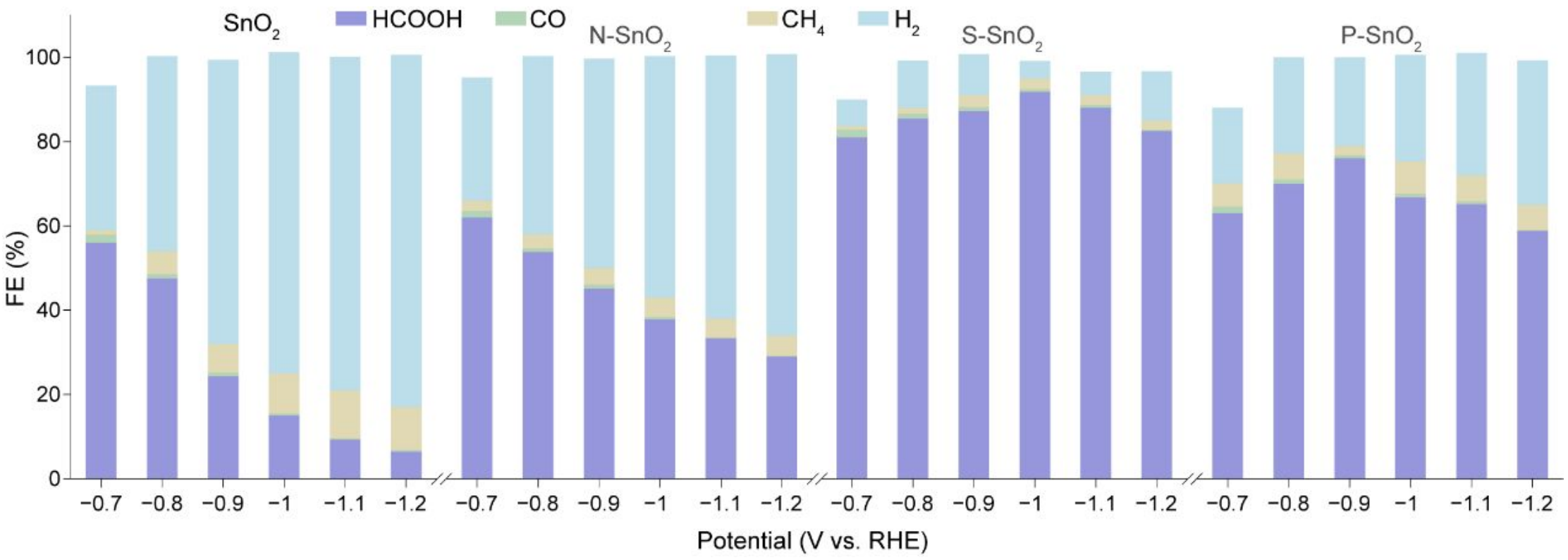

Supplementary Fig. 6 | Faradaic efficiencies of gaseous and liquid products over SnO_2-based catalysts. Faradaic efficiencies (FEs) of HCOOH, CO, CH_4, and H_2 over SnO_2, N-SnO_2, S-SnO_2, and P-SnO_2 measured in a flow-cell reactor.

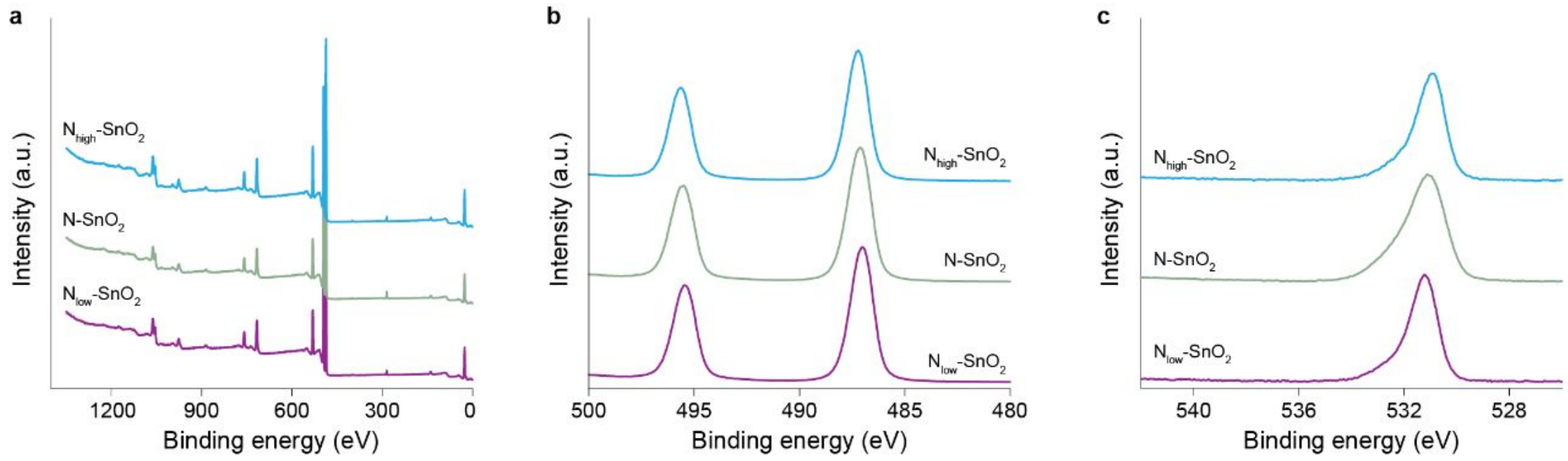

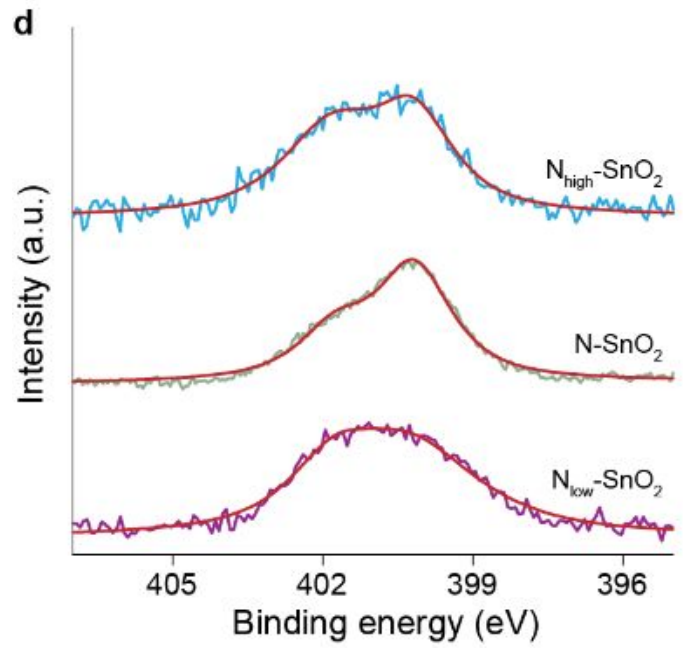

e

	Name	Start E	End BE	Height CPS	FWHM eV	Area (P) CPS. eV	Area (N) TPP-2M	Atomic %
N_{low}-SnO_2	Sn 3d	500.20	480.15	69184.52	1.33	868427.36	18586.34	32.76
	O 1s	536.50	526.50	91637.48	1.20	739628.84	37289.51	66.48
	N 1s	405.50	395.50	1286.47	1.57	8627.35	426.71	0.76
N-SnO_2	Sn 3d	500.20	480.15	66028.35	1.37	832695.48	17768.92	31.54
	O 1s	536.50	526.50	92984.16	1.25	754382.69	37493.58	66.55
	N 1s	405.50	395.50	3024.67	1.46	18964.27	1075.36	1.91
N_{high}-SnO_2	Sn 3d	500.20	480.15	63751.83	1.40	798624.51	17042.86	30.29
	O 1s	536.50	526.50	94426.59	1.29	773615.42	37516.49	66.62
	N 1s	405.50	395.50	4816.25	1.62	30284.76	1740.25	3.09

Supplementary Fig. 7 | XPS characterization and dopant quantification of the N-doped SnO_2 series (N_{low}-SnO_2, N-SnO_2, and N_{high}-SnO_2). (a) XPS survey spectra, (b) High-resolution Sn 3*d* spectra and (c) O 1*s* spectra, (d) High-resolution N 1*s* spectra, (e) Peak-integration table summarizing the extracted N atomic percentages.

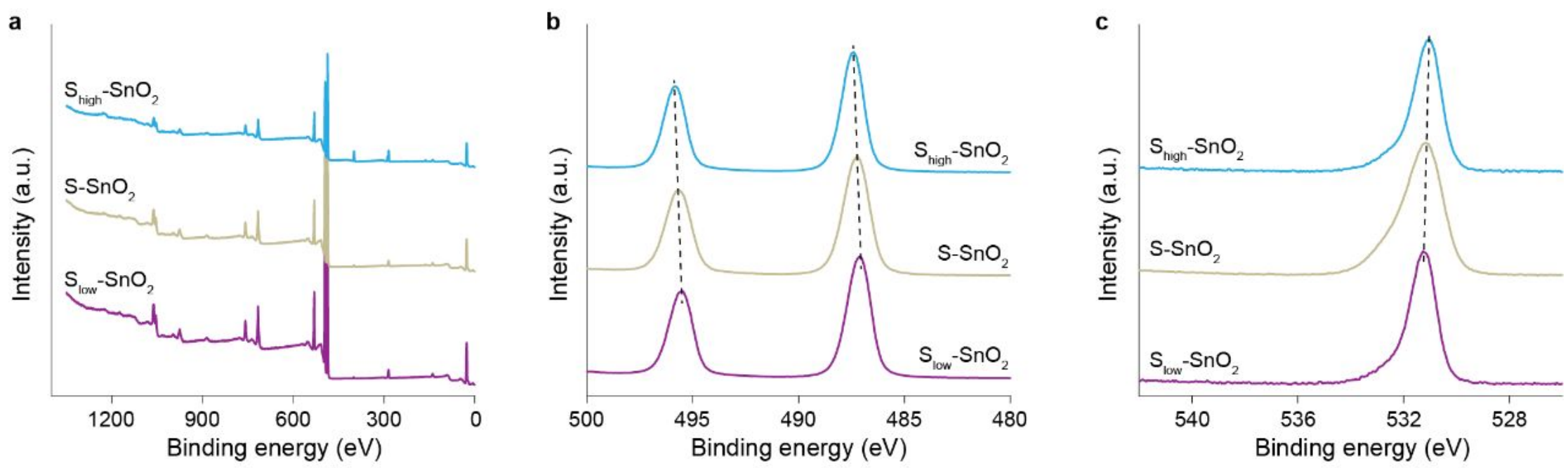

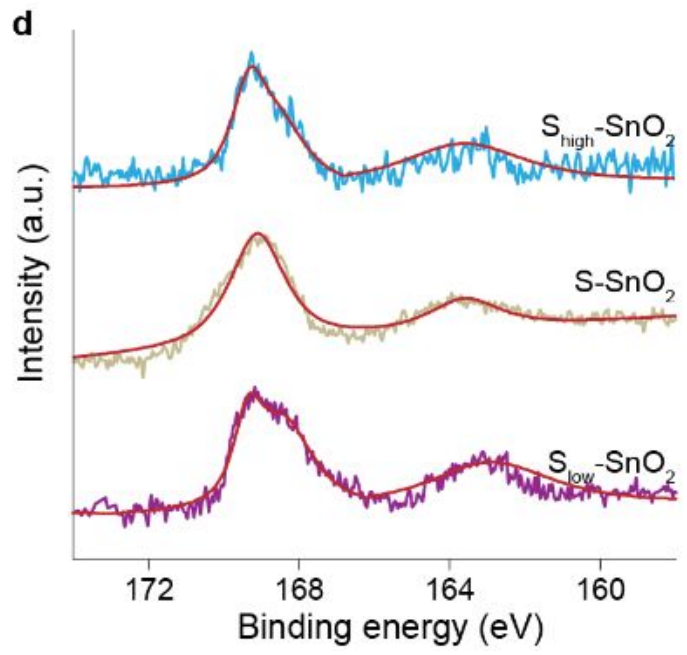

e

	Name	Start E	End BE	Height CPS	FWHM eV	Area (P) CPS. eV	Area (N) TPP-2M	Atomic %
S_{low}-SnO_2	Sn 3d	500.20	480.15	69184.52	1.33	871526.34	18642.71	32.91
	O 1s	536.50	526.50	91637.48	1.19	734928.66	37135.84	66.21
	S 2p	172.50	158.50	1286.47	1.27	9563.28	493.62	0.88
S-SnO_2	Sn 3d	500.20	480.15	66028.35	1.36	836274.51	17831.46	31.62
	O 1s	536.50	526.50	92984.16	1.24	752641.39	37405.28	66.32
	S 2p	172.50	158.50	3024.67	1.35	23186.74	1162.57	2.06
S_{high}-SnO_2	Sn 3d	500.20	480.15	63751.83	1.40	802947.26	17126.93	30.24
	O 1s	536.50	526.50	94426.59	1.28	771538.42	37398.65	66.39
	S 2p	172.50	158.50	4816.25	1.28	37192.85	1897.34	3.37

Supplementary Fig. 8 | XPS characterization and dopant quantification of the S-doped SnO_2 series (S_{low}-SnO_2, S-SnO_2, and S_{high}-SnO_2). (a) XPS survey spectra, (b) High-resolution Sn 3*d* spectra and (c) O 1*s* spectra, (d) High-resolution S 2*p* spectra, (e) Peak-integration table summarizing the extracted S atomic percentages.

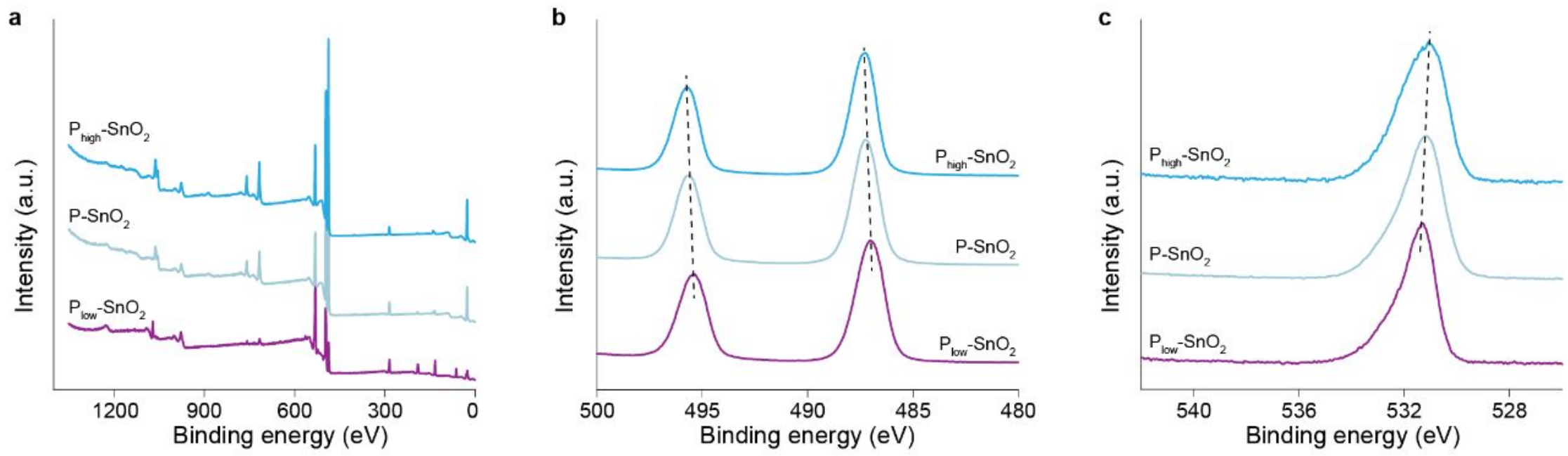

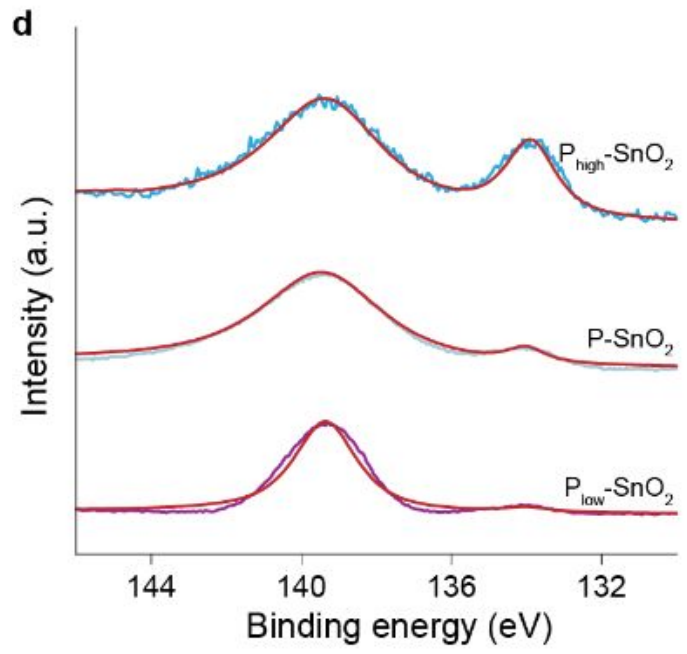

e

	Name	Start E	End BE	Height CPS	FWHM eV	Area (P) CPS. eV	Area (N) TPP-2M	Atomic %
P_{low}-SnO_2	Sn 3d	500.20	480.15	68425.37	1.34	862314.58	18472.25	32.84
	O 1s	536.50	526.50	92418.66	1.21	742685.91	37246.82	66.21
	P 2p	144.50	128.50	1462.83	1.16	10284.47	535.21	0.95
P-SnO_2	Sn 3d	500.20	480.15	65173.49	1.38	823946.25	17654.79	31.47
	O 1s	536.50	526.50	93582.14	1.26	758391.63	37235.61	66.36
	P 2p	144.50	128.50	3276.59	1.23	24618.72	1218.64	2.17
P_{high}-SnO_2	Sn 3d	500.20	480.15	62946.81	1.41	796725.43	16982.13	30.18
	O 1s	536.50	526.50	94173.28	1.29	769428.36	37324.89	66.33
	P 2p	144.50	128.50	4928.74	1.31	38276.85	1964.58	3.49

Supplementary Fig. 9 | XPS characterization and dopant quantification of the P-doped SnO_2 series (P_{low}-SnO_2, P-SnO_2, and P_{high}-SnO_2). (a) XPS survey spectra, (b) High-resolution Sn 3*d* spectra and (c) O 1*s* spectra, (d) High-resolution P 2*p* spectra, (e) Peak-integration table summarizing the extracted P atomic percentages.

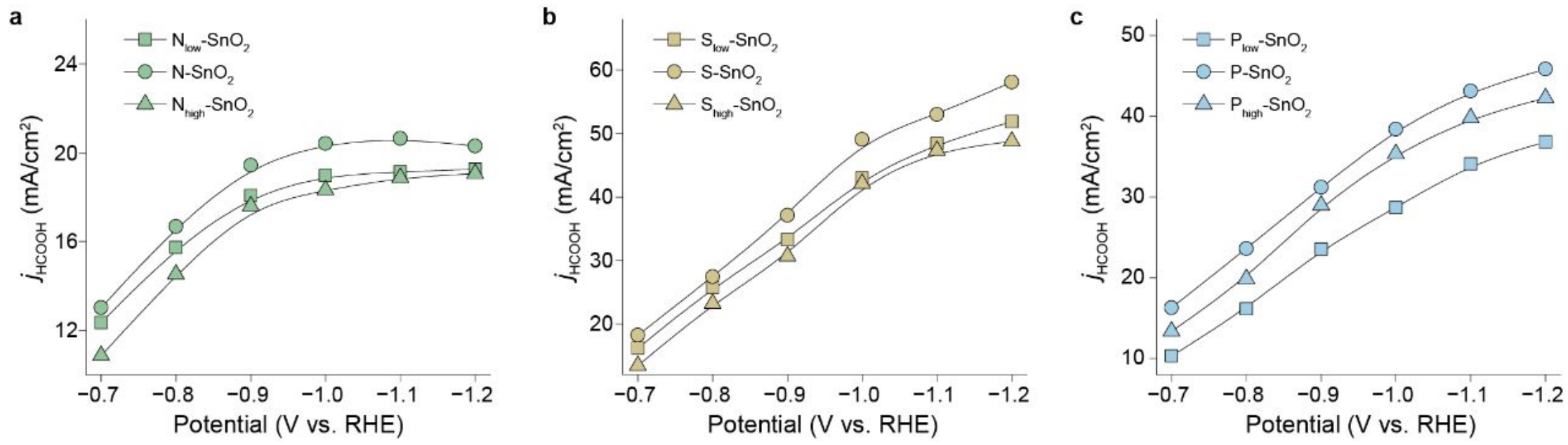

Supplementary Fig. 10 | Dopant-level-dependent formate partial current densities (j_{HCOOH}) for the N-, S-, and P-doped SnO_2 series. The j_{HCOOH} as a function of potential (-0.7 to -1.2 V vs. RHE) for catalysts with graded dopant contents prepared, (a) N_{low}-SnO_2, N-SnO_2, and N_{high}-SnO_2; (b) S_{low}-SnO_2, S-SnO_2, and S_{high}-SnO_2; and (c) P_{low}-SnO_2, P-SnO_2, and P_{high}-SnO_2.

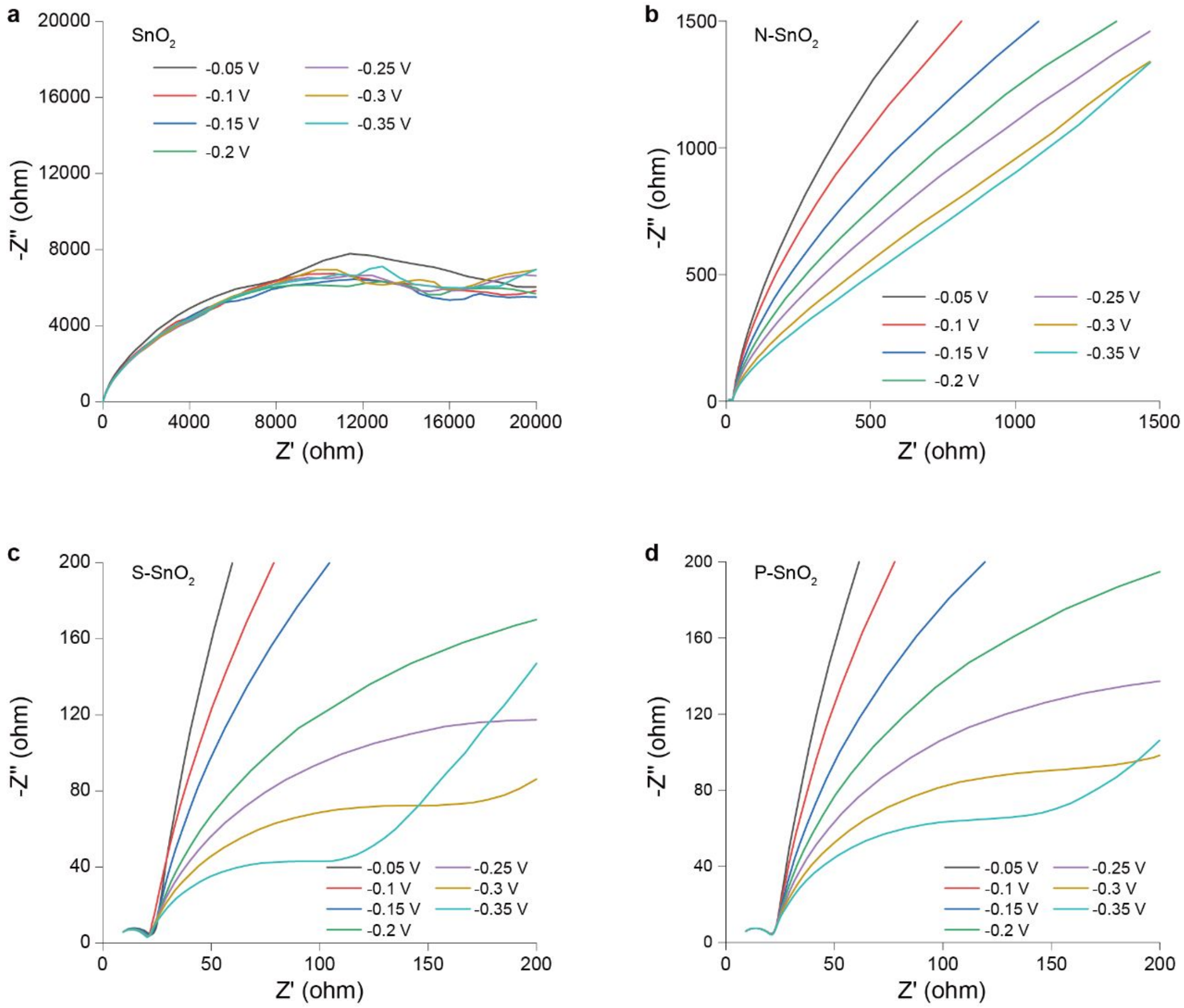

Supplementary Fig. 11 | Nyquist plots (EIS) of SnO_2 and heteroatom-doped SnO_2 electrodes. EIS spectra collected at the indicated applied potentials (-0.05 to -0.35 V) for (a) SnO_2, (b) N-SnO_2, (c) S-SnO_2, and (d) P-SnO_2.

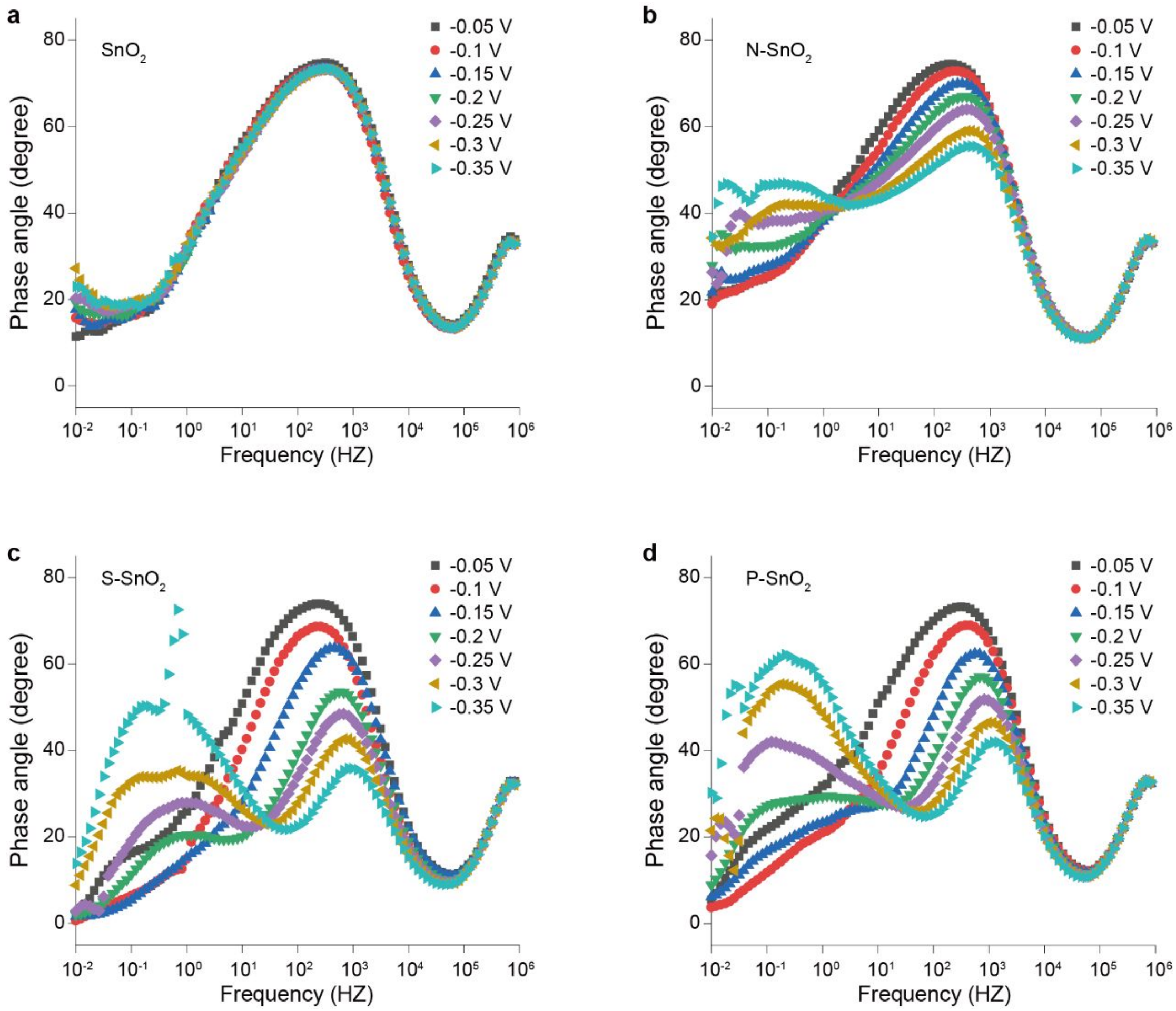

Supplementary Fig. 12 | Phase-angle (Bode) plots corresponding to the EIS measurements in Figure R1-14. Phase angle as a function of frequency for (a) SnO_2, (b) N-SnO_2, (c) S-SnO_2, and (d) P-SnO_2 measured at the indicated applied potentials (−0.05 to −0.35 V).

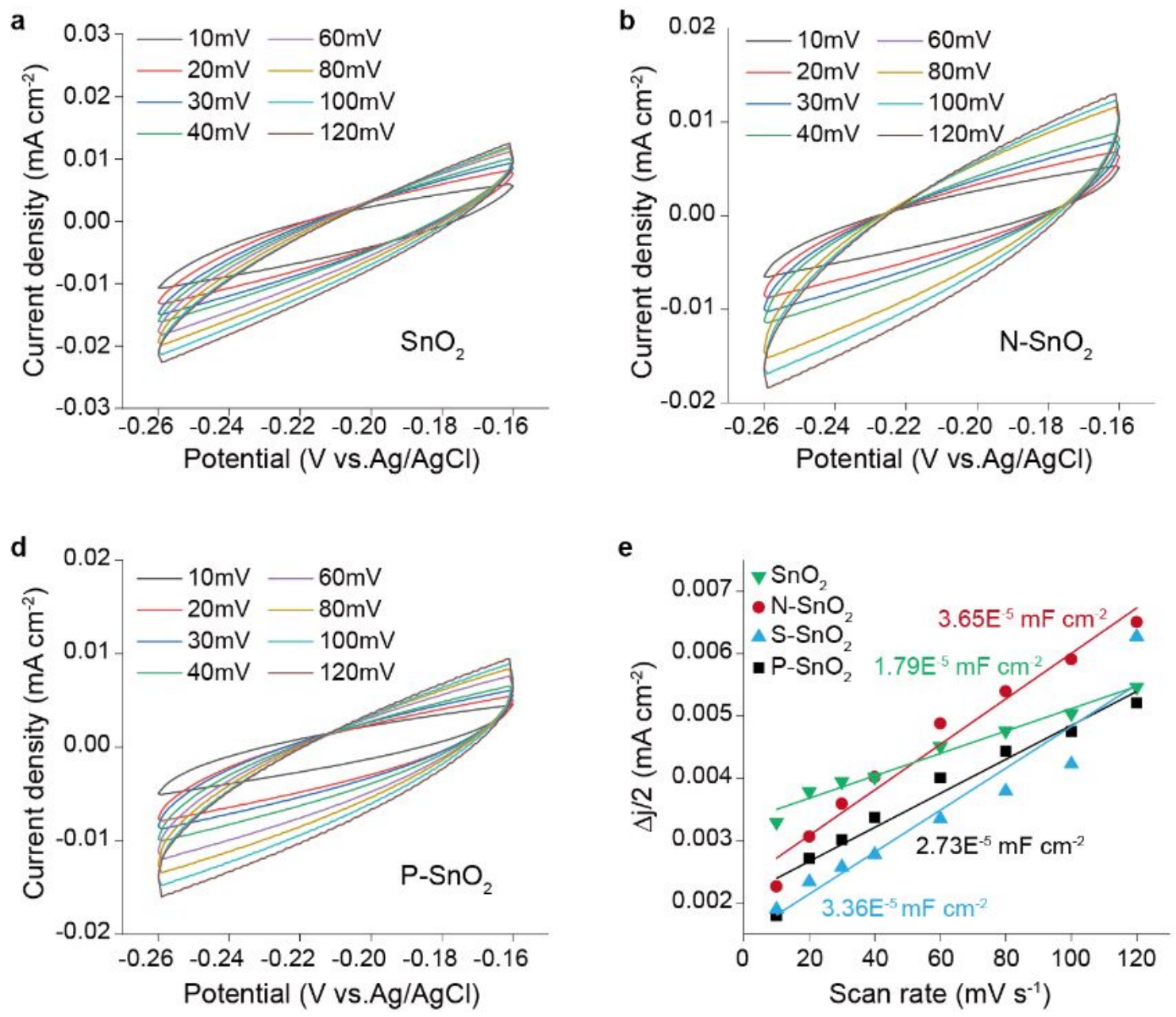

Supplementary Fig. 13 | Double-layer capacitance (Cdl) measurements for estimating ECSA of SnO_2 and doped SnO_2 electrodes. Non-faradaic cyclic voltammograms recorded at different scan rates (10-120 mV s^{-1}) for (a) SnO_2, (b) N-SnO_2, (c) S-SnO_2, and (d) P-SnO_2 in the non-faradaic potential window. (e) The corresponding Δj/2 versus scan rate plots used to extract Cdl (slopes), yielding Cdl values of 2.73 mF cm^{-2} (SnO_2), 1.79 mF cm^{-2} (N-SnO_2), 3.36 mF cm^{-2} (S-SnO_2), and 3.65 mF cm^{-2} (P-SnO_2).

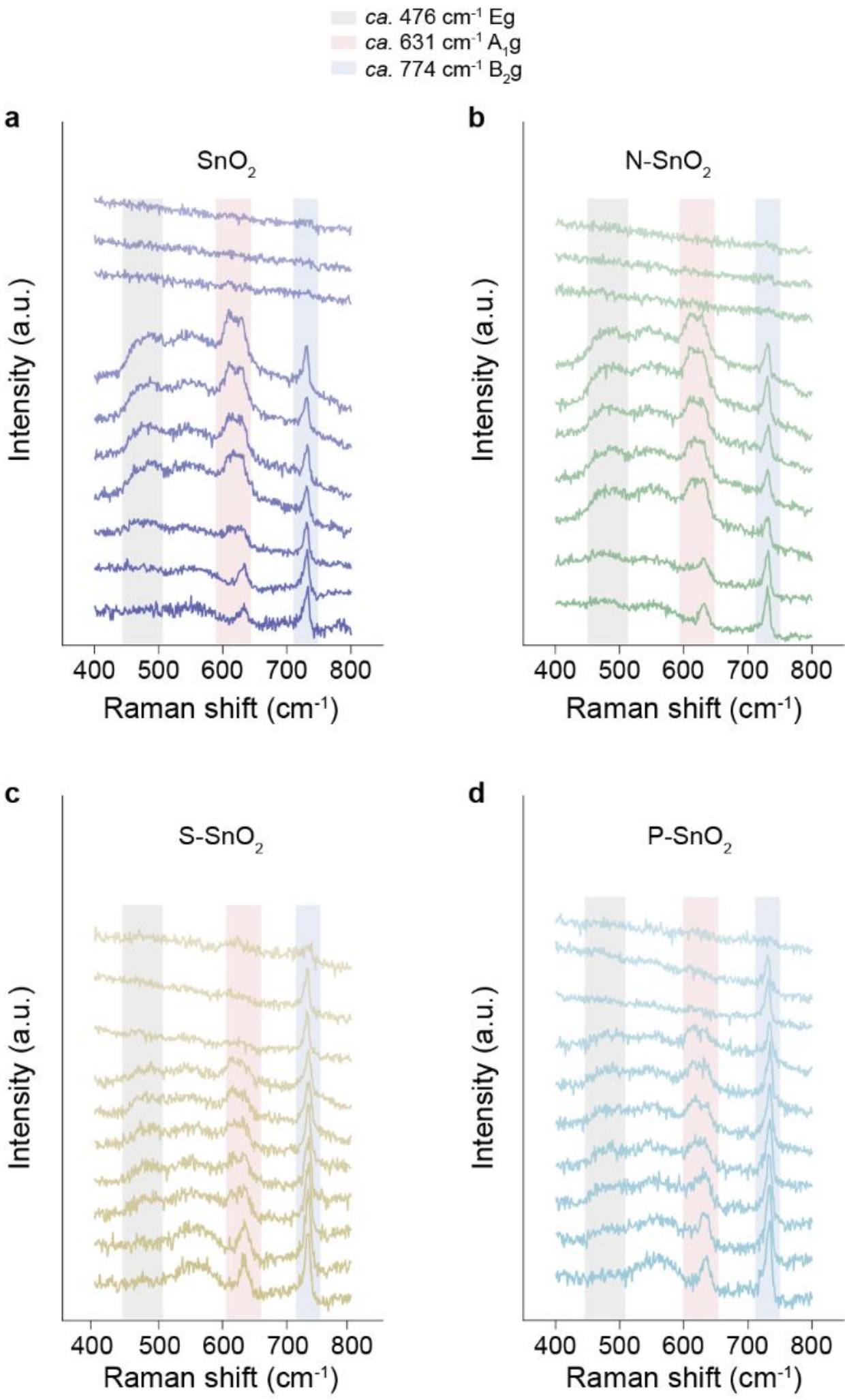

Supplementary Fig. 14 | *In-situ* Raman spectra of pristine and heteroatom-doped SnO_2 catalysts under cathodic potentials. Potential-dependent Raman spectra of (a) SnO_2, (b) N-SnO_2, (c) S-SnO_2, and (d) P-SnO_2 collected during CO_2RR. The characteristic lattice phonon modes of rutile SnO_2, Eg (~476 cm^{-1}), A1g (~631 cm^{-1}), and B2g (~774 cm^{-1}), are highlighted. Pristine SnO_2 and N-SnO_2 exhibit pronounced peak attenuation and broadening under increasing cathodic bias, indicative of progressive Sn-O bond disruption and structural reduction.

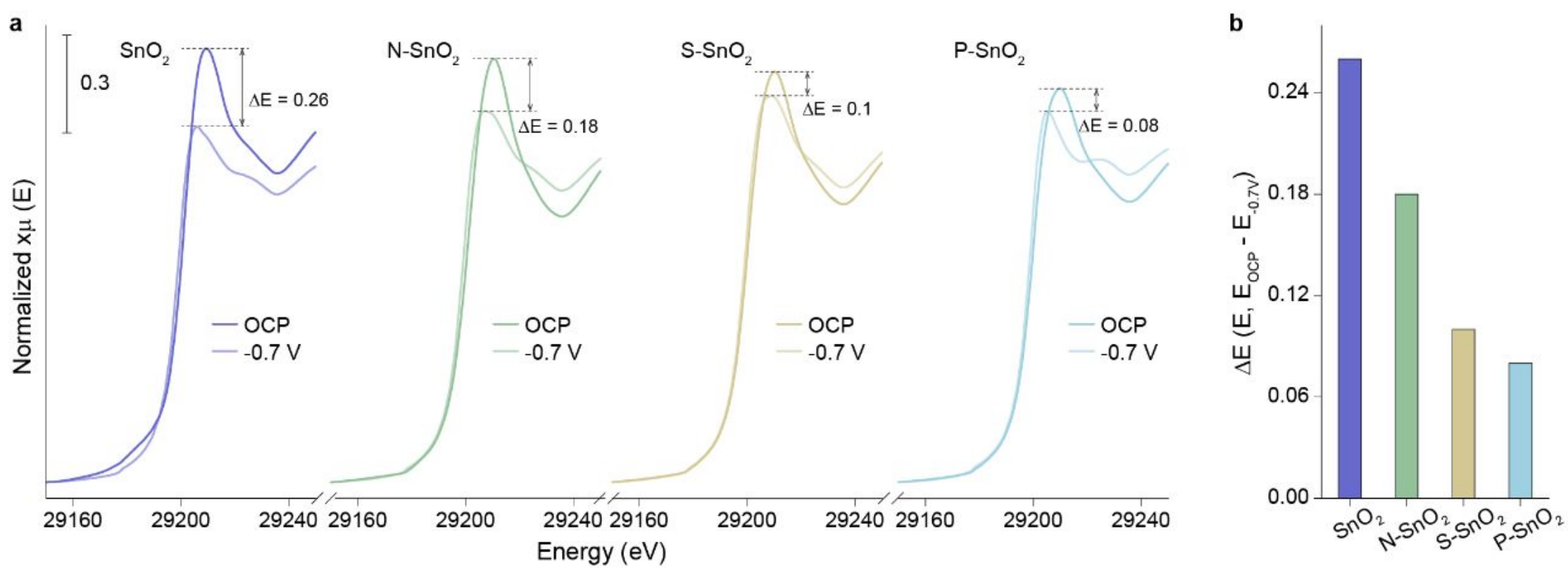

Supplementary Fig. 15. Operando Sn L_3-edge XANES revealing the dynamic electronic response under CO_2RR conditions. (a) Sn L_3-edge XANES spectra of SnO_2, N-SnO_2, S-SnO_2, and P-SnO_2 collected at open-circuit potential (OCP) and -0.7 V vs. RHE. All samples exhibit a decrease in white-line intensity under bias, indicative of partial reduction of Sn^{4+}. (b) Quantified edge shifts (ΔE) extracted from panel a. Pristine SnO_2 shows the largest energy shift (0.26 eV), whereas doped samples display progressively suppressed electronic responses, with P-SnO_2 and S-SnO_2 exhibiting the smallest ΔE values.

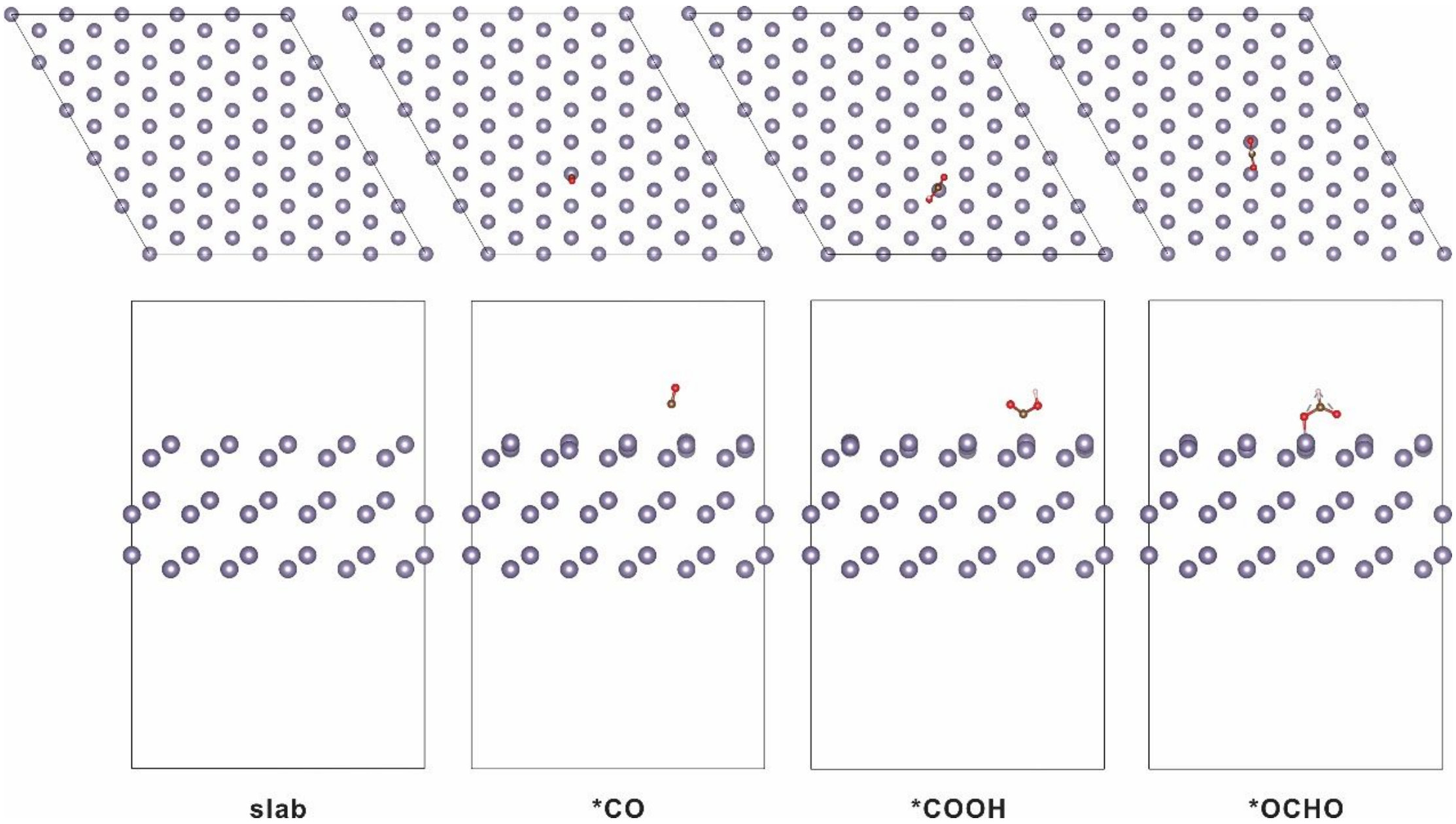

Supplementary Fig. 16 | Optimized adsorption configurations of CO_2 reduction intermediates on SnO_2. Top and side views of the optimized structures for pristine SnO_2 slab and key CO_2RR intermediates (*CO, *COOH, and *OCHO) obtained from DFT calculations. The adsorption geometries illustrate distinct binding motifs, where *CO binds in a monodentate configuration, while *COOH and *OCHO exhibit different coordination modes on surface Sn sites.

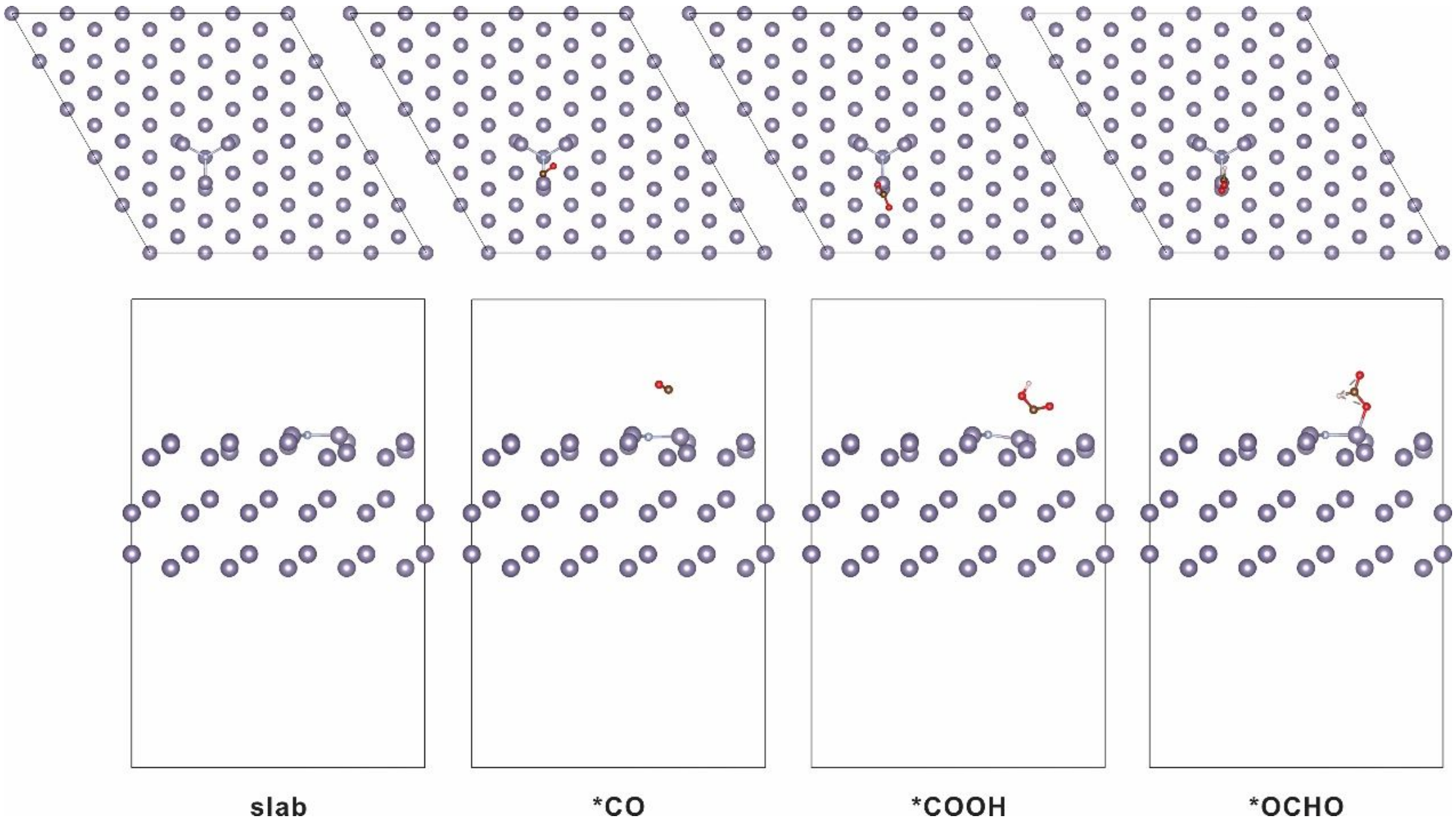

Supplementary Fig. 17 | Optimized adsorption configurations of CO_2 reduction intermediates on N-SnO_2. Top and side views of the optimized slab model and key intermediates (*CO, *COOH, and *OCHO) adsorbed on N-SnO_2 obtained from DFT calculations. Compared with pristine SnO_2, nitrogen incorporation modifies the local coordination environment and electronic distribution of surface Sn sites, leading to altered adsorption geometries and binding configurations of reaction intermediates.

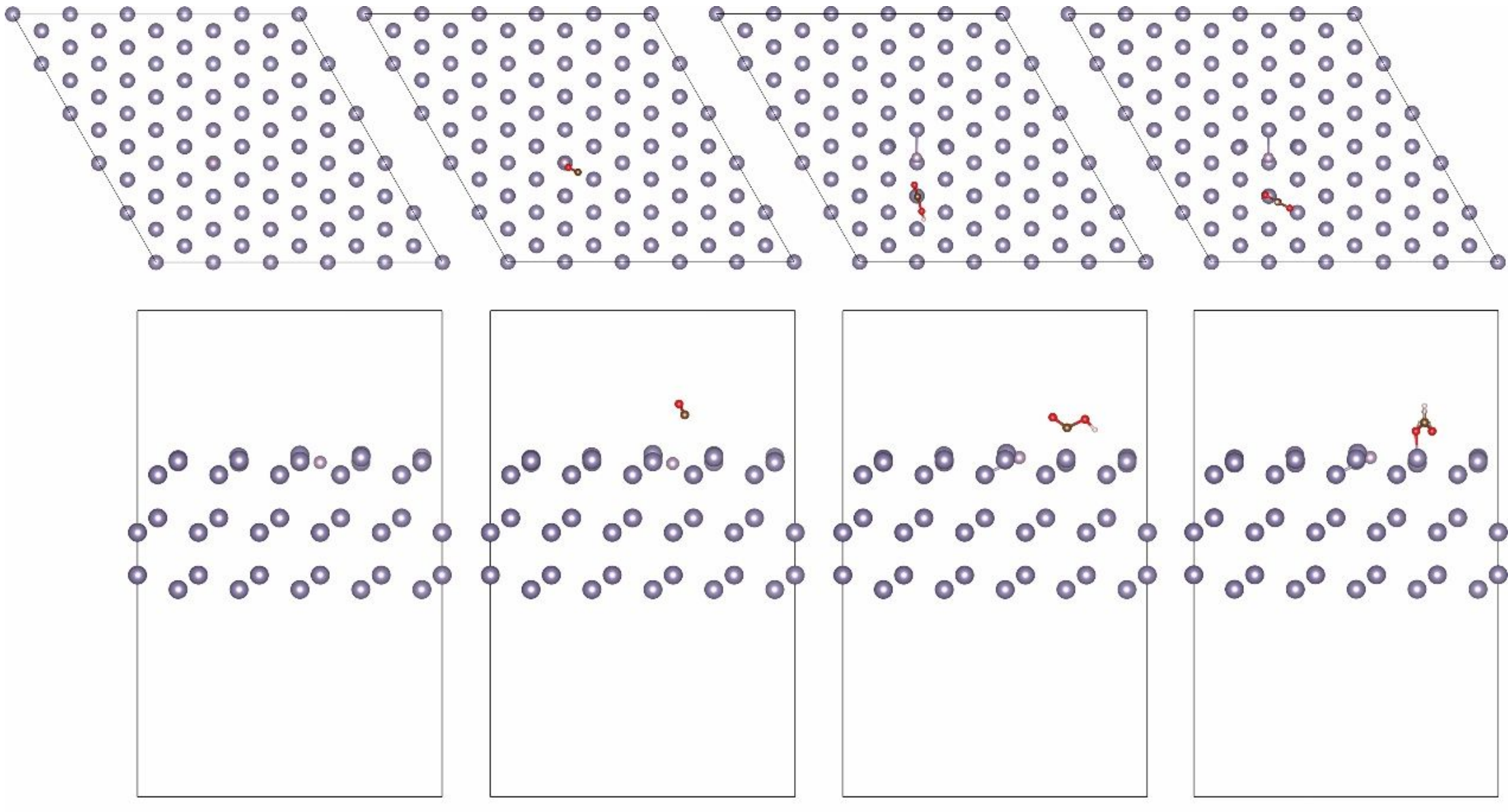

Supplementary Fig. 18 | Optimized adsorption configurations of CO_2 reduction intermediates on P-SnO_2. Top and side views of the optimized slab model and key CO_2RR intermediates (*CO, *COOH, and *OCHO) adsorbed on P-SnO_2 derived from DFT calculations. Phosphorus incorporation modifies the surface electronic structure and coordination environment, leading to altered adsorption of geometries.

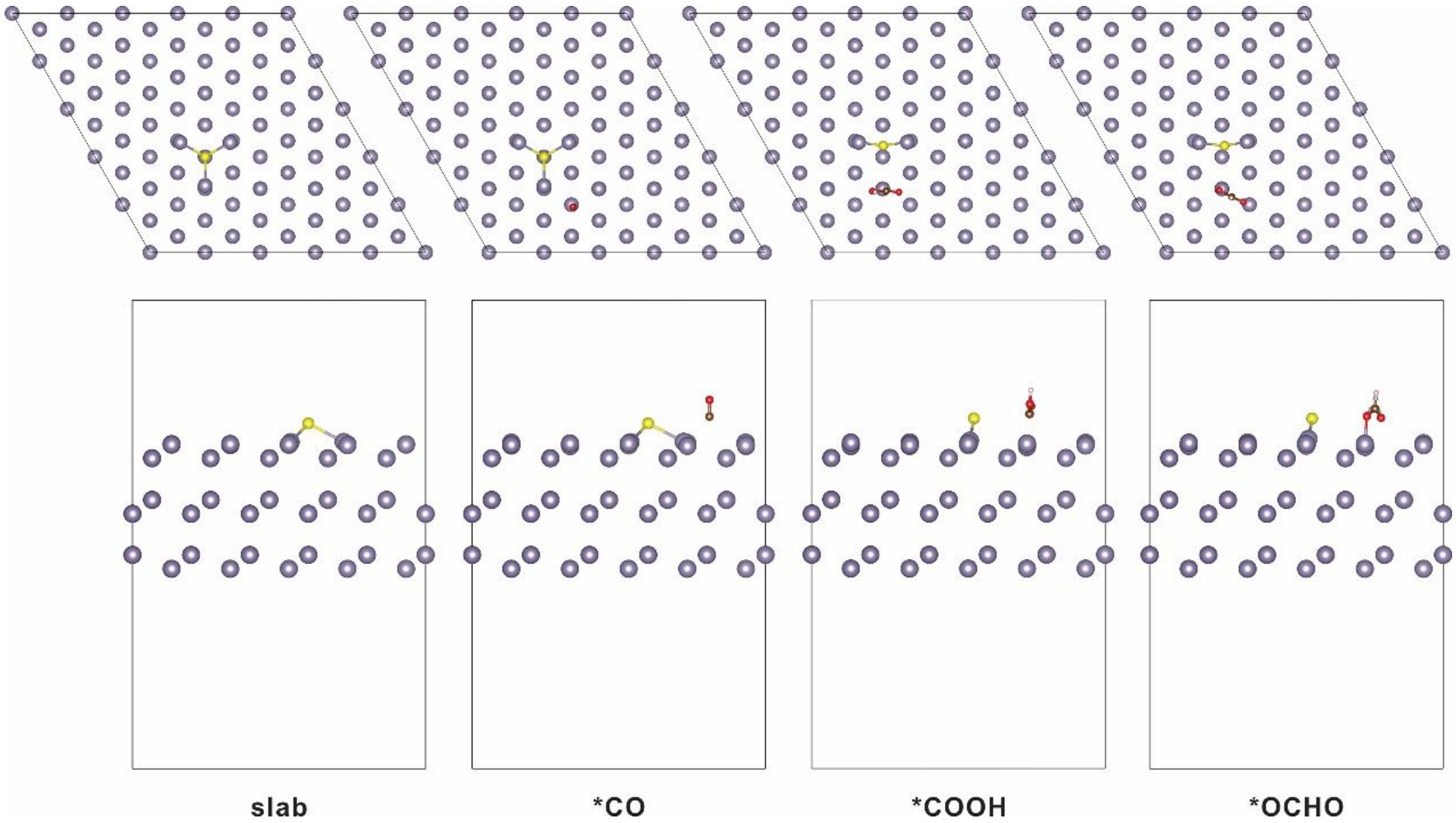

Supplementary Fig. 19 | Optimized adsorption configurations of CO_2 reduction intermediates on S-SnO_2. Top and side views of the optimized slab model and key CO_2RR intermediates (*CO, *COOH, and *OCHO) adsorbed on S-SnO_2 obtained from DFT calculations. Sulfur incorporation induces pronounced local electronic polarization around neighboring Sn sites, leading to distinct adsorption geometries compared with pristine and P-doped surfaces.

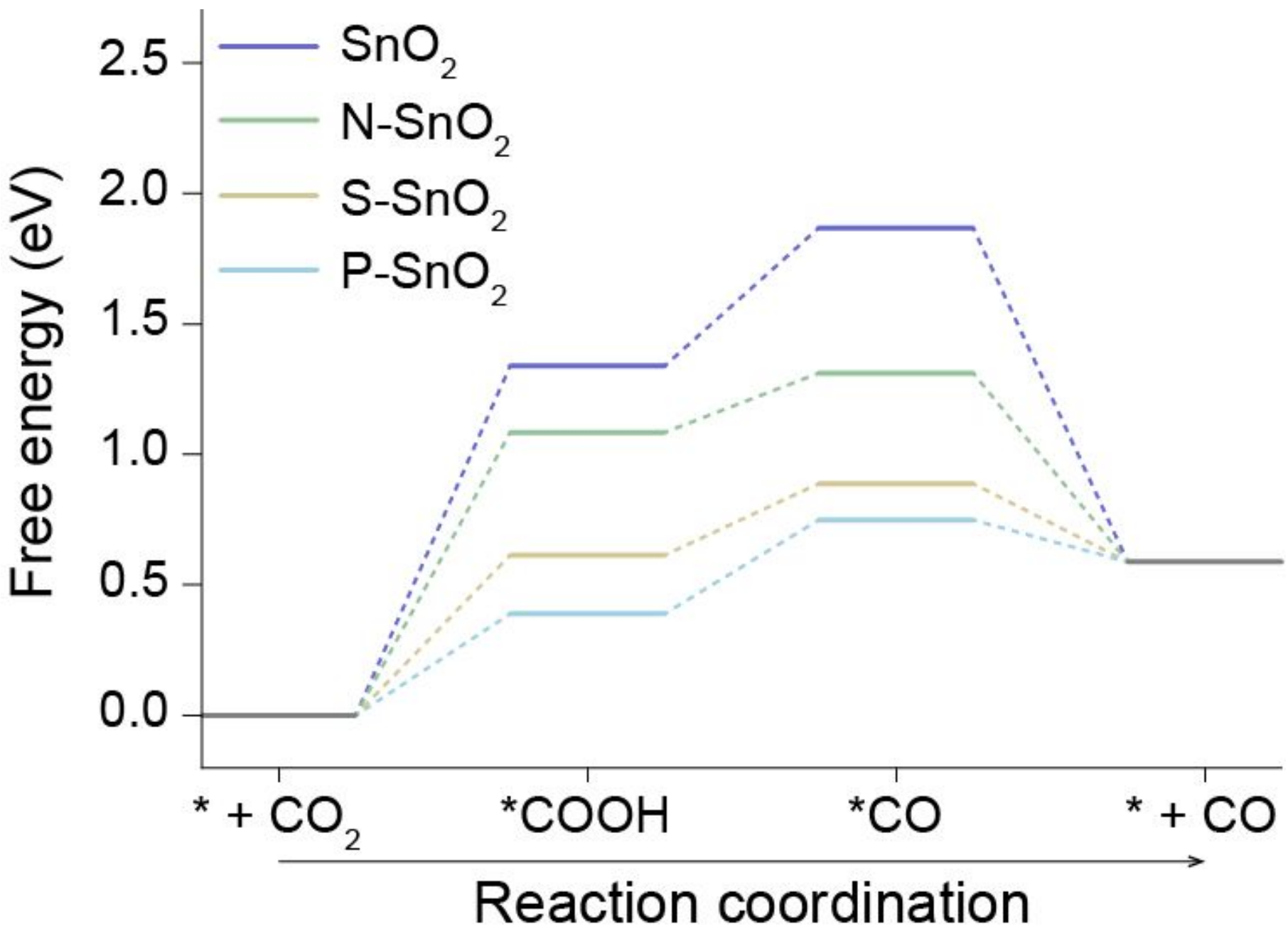

Supplementary Fig. 20 | Free energy profile for CO production via *COOH.

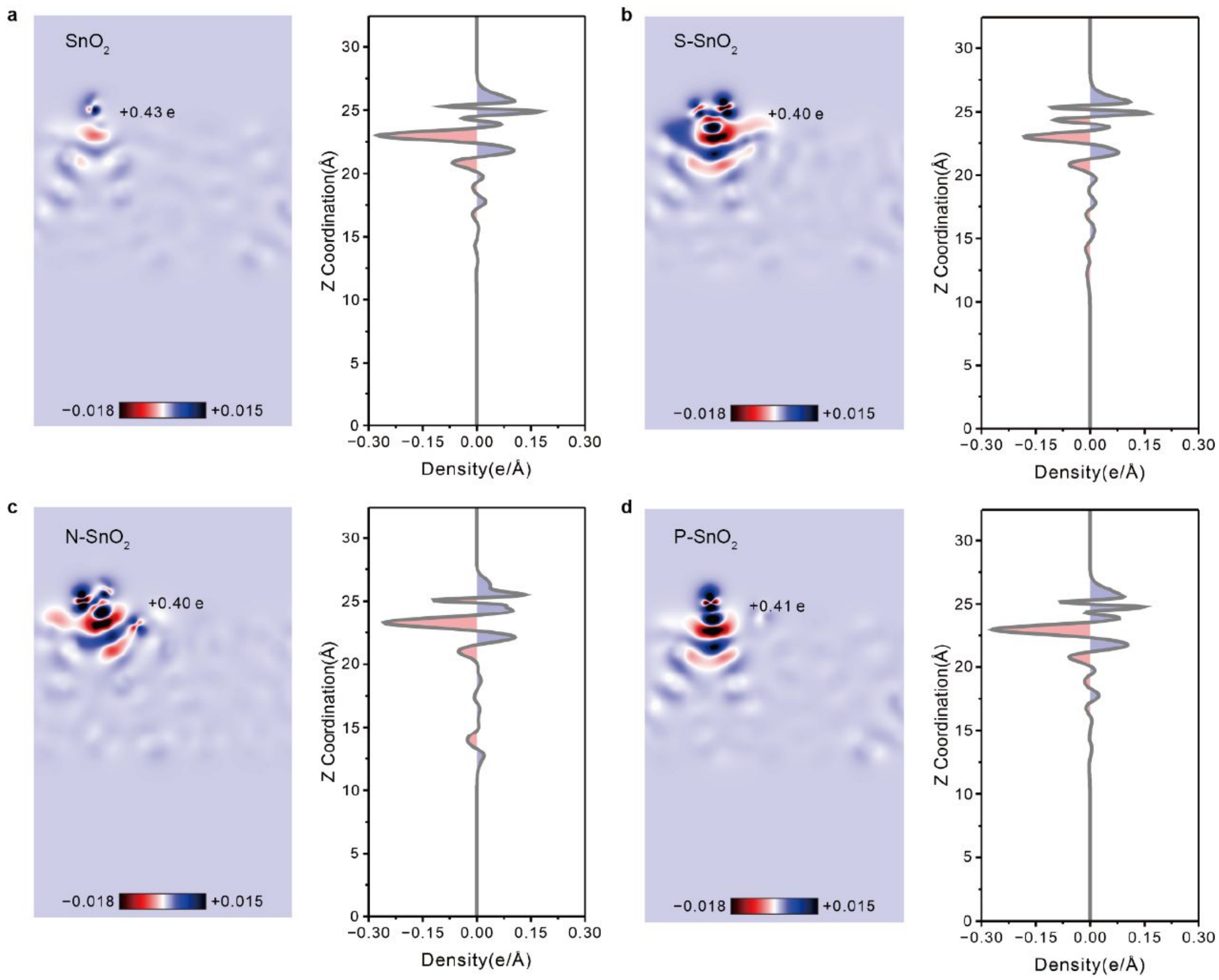

Supplementary Fig. 21 | Differential charge density and planar-averaged charge distribution for the *COOH intermediate on SnO_2-based catalysts. Differential charge density plots (left) and corresponding planar-averaged charge density profiles along the surface normal (right) for the *COOH intermediate adsorbed on (a) SnO_2, (b) S-SnO_2, (c) N-SnO_2, and (d) P-SnO_2. Charge accumulation and depletion are represented by red and blue regions, respectively. Heteroatom incorporation induces pronounced electron redistribution around the adsorption site, as reflected by the interfacial charge polarization and net charge transfer values.

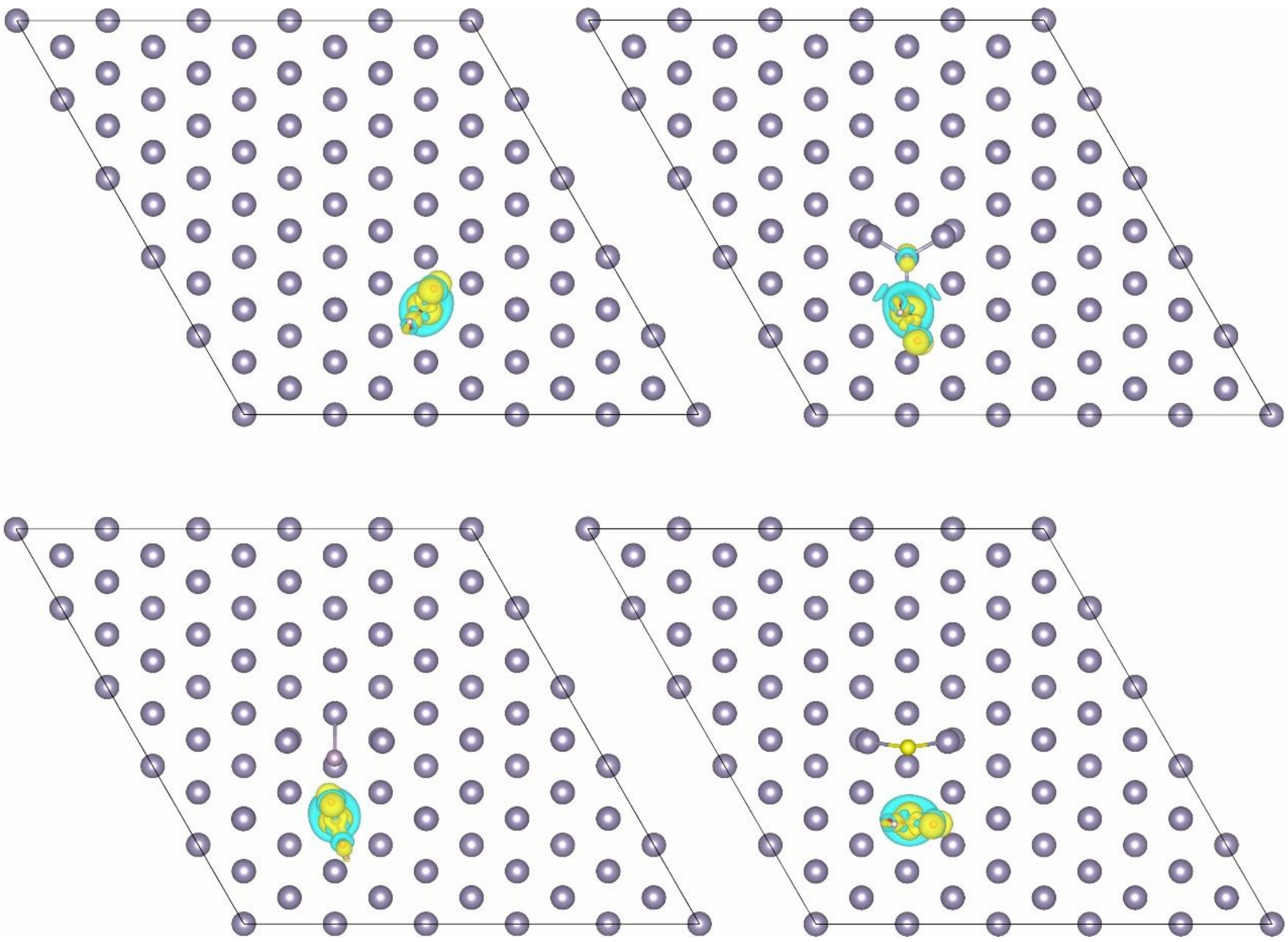

Supplementary Fig. 22 | Three-dimensional differential charge density of the *COOH intermediate on SnO_2-based catalysts. Isosurface plots of differential charge density for the *COOH intermediate adsorbed on pristine SnO_2 and heteroatom-doped surfaces. Yellow and cyan regions represent charge accumulation and depletion, respectively. Heteroatom incorporation induces pronounced interfacial charge polarization around the adsorption site, reflecting modified electronic coupling between *COOH and surface Sn centers.

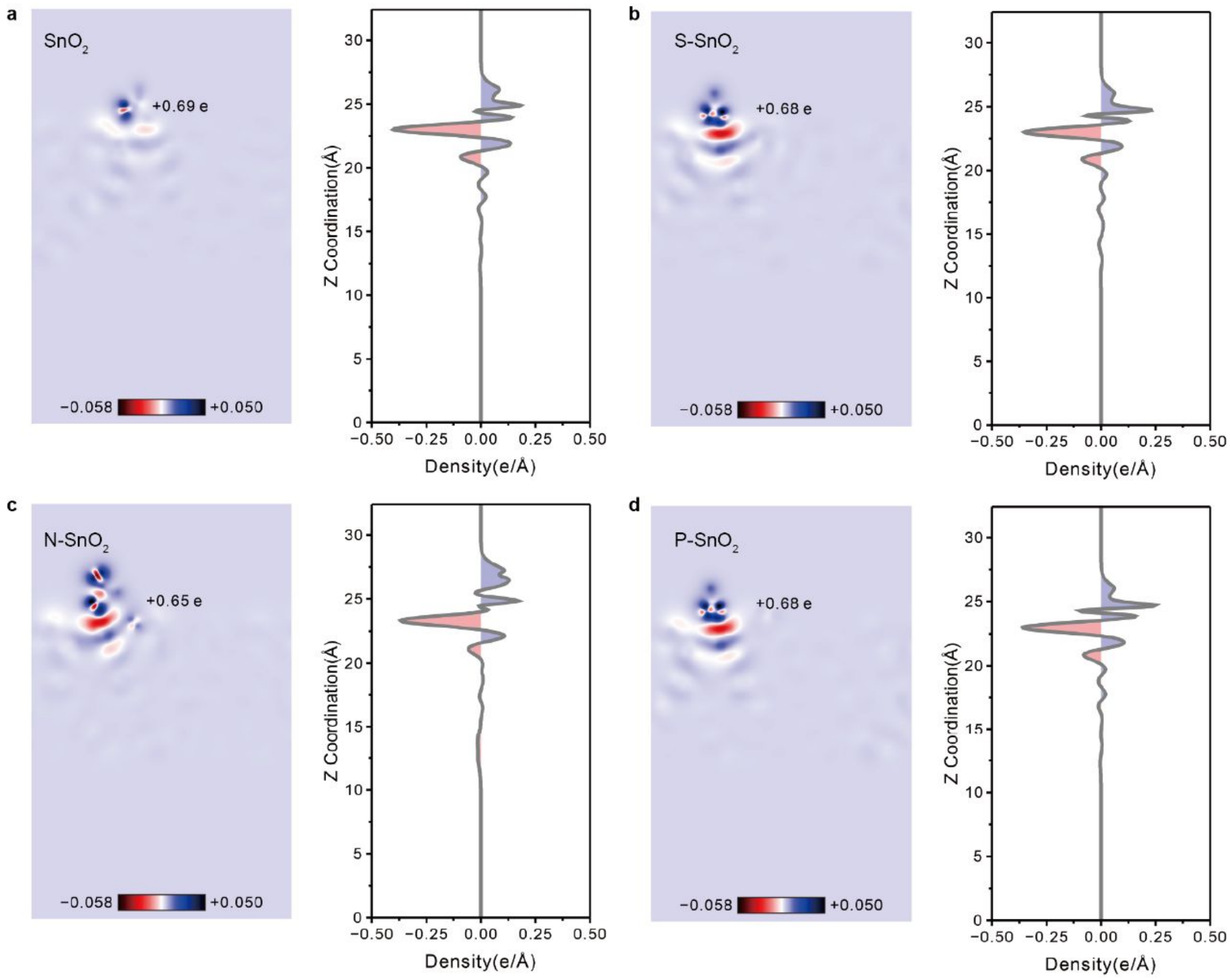

Supplementary Fig. 23 | Differential charge density and planar-averaged charge distribution for the OCHO intermediate on SnO_2-based catalysts. Differential charge density plots (left) and corresponding planar-averaged charge density profiles along the surface normal (right) for the *OCHO intermediate adsorbed on (a) SnO_2, (b) S-SnO_2, (c) N-SnO_2, and (d) P-SnO_2. Charge accumulation and depletion are represented by red and blue regions, respectively. All systems exhibit substantial interfacial charge polarization upon *OCHO adsorption, with comparable net charge transfer values.

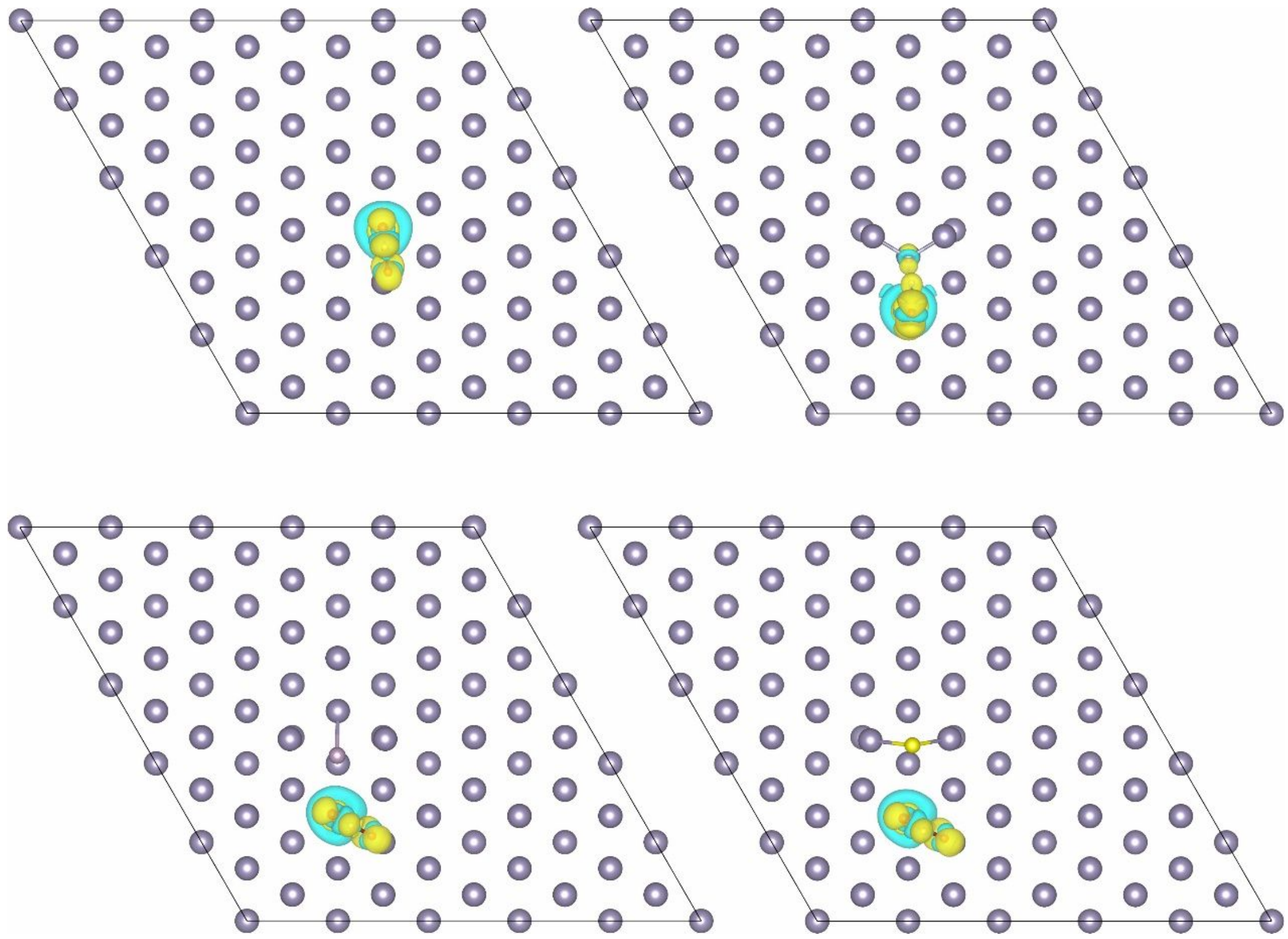

Supplementary Fig. 24 | Three-dimensional differential charge density of the OCHO intermediate on SnO_2-based catalysts. Isosurface plots of differential charge density for the *OCHO intermediate adsorbed on pristine and heteroatom-doped SnO_2 surfaces. Yellow and cyan regions denote charge accumulation and depletion, respectively. Heteroatom incorporation induces noticeable interfacial charge polarization around the adsorption site; however, the magnitude and spatial distribution of charge redistribution remain broadly comparable across the different catalysts.

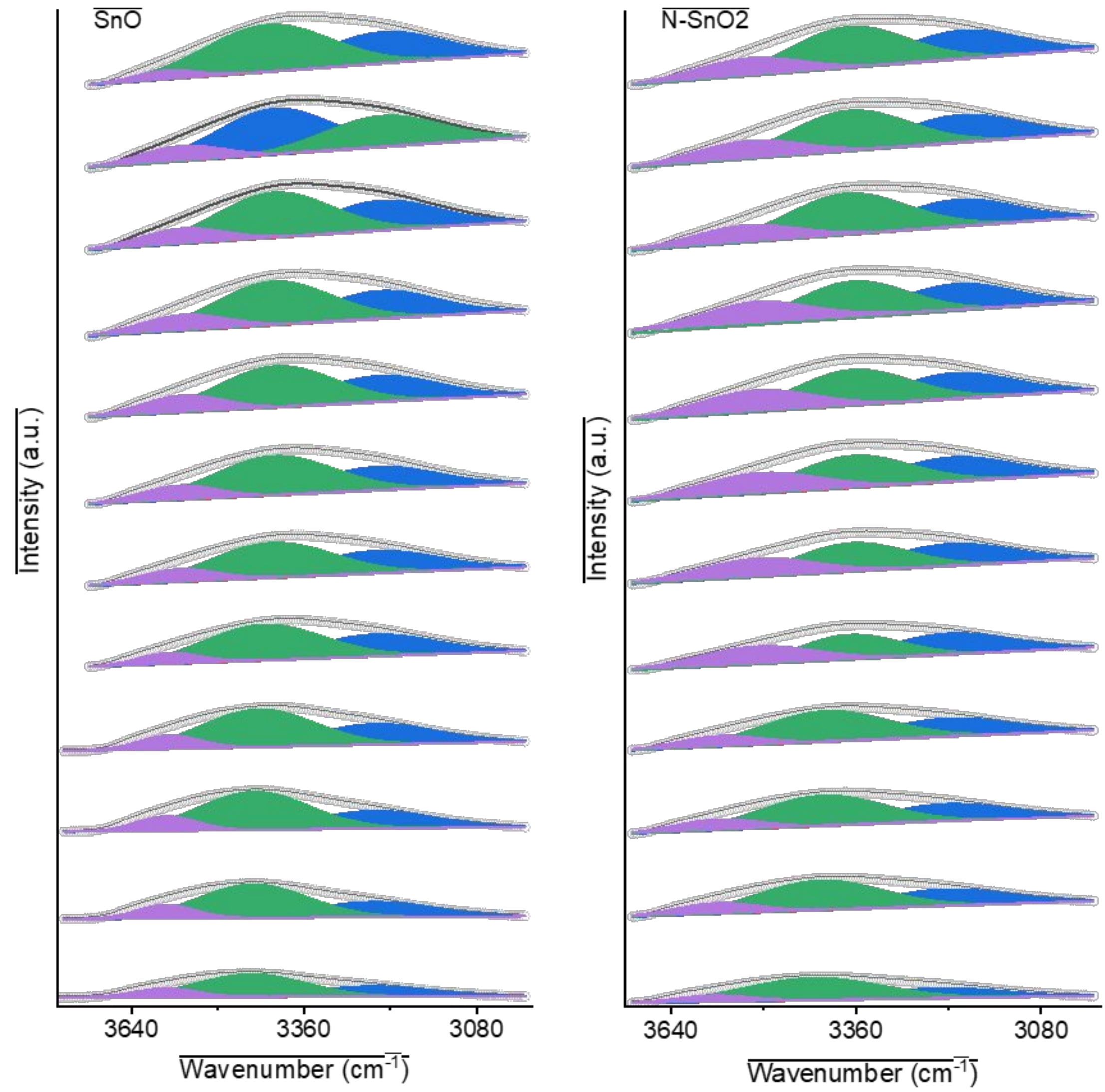

Supplementary Fig. 25 | Dopant-mediated restructuring of the interfacial H_2O network. *Operando* ATR-SEIRAS spectra in the O-H stretching region (3080-3640 cm^{-1}) recorded under progressively increasing cathodic potentials for SnO_2 (a) and N-SnO_2 (b). Deconvolution of the broad O-H envelope reveals distinct evolution of hydrogen-bonding populations upon bias.

The operando ν_{OH} envelope (3080-3640 cm^{-1}) is discussed in terms of distinct hydrogen-bonding environments commonly used for electrified interfaces: strongly hydrogen-bonded (ice-like), weakly hydrogen-bonded / H-bond-disrupted, and an optional free-OH-like high-frequency shoulder. The purpose of the deconvolution is to quantify the relative evolution of hydrogen-bonding populations under bias across catalysts; molecular orientation is evaluated independently by AIMD angular statistics (cosθ maps, **Fig. S26**) and should not be inferred solely from ν_{OH} fitting because ATR-SEIRAS intensities depend on dipole orientation (approximately $\cos^2\theta$).

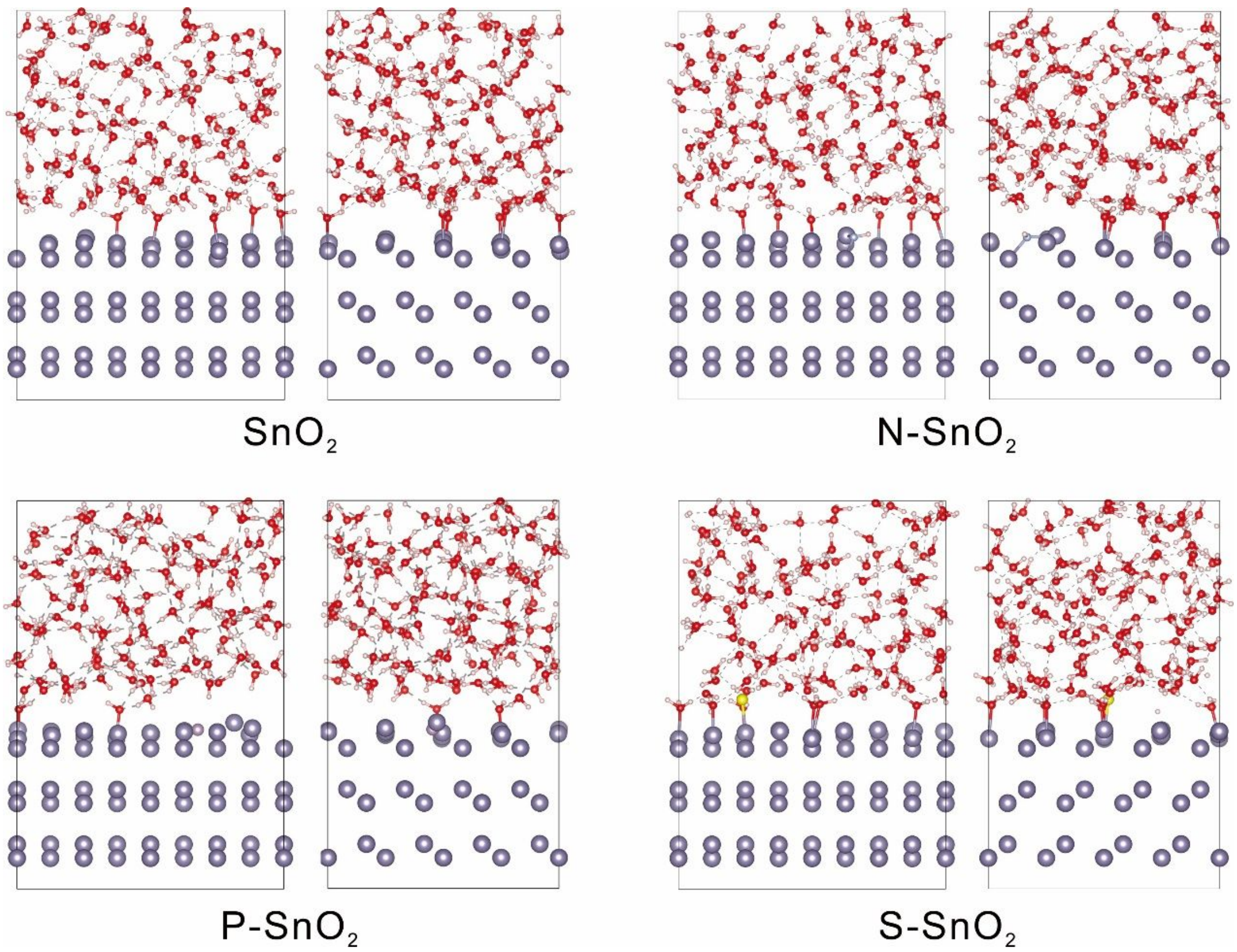

Supplementary Fig. 26 | AIMD snapshots of interfacial H_2O configurations on SnO_2-based catalysts. Representative ab initio molecular dynamics (AIMD) snapshots of the solid-liquid interface for pristine SnO_2 and N-, P-, and S-doped SnO_2 surfaces.

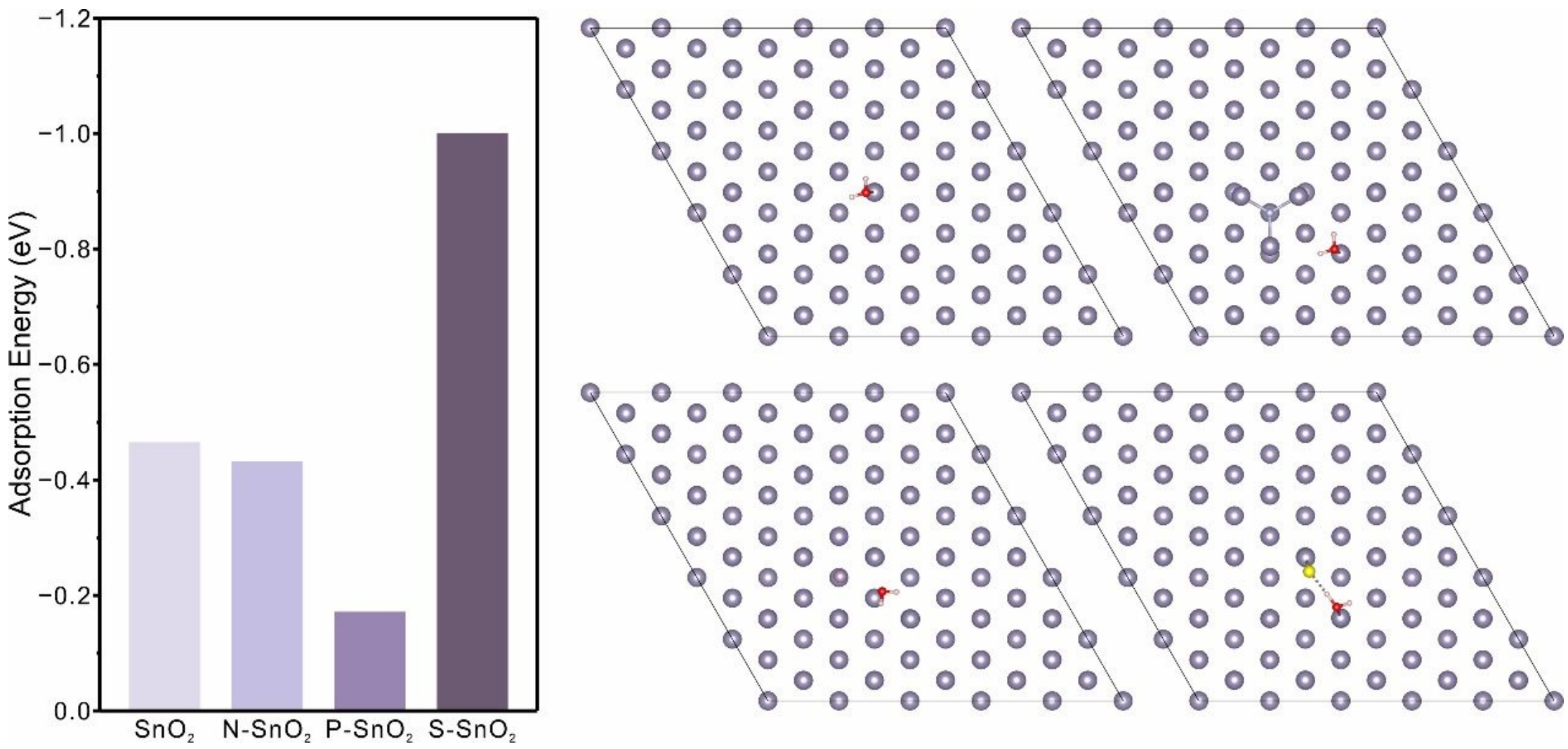

Supplementary Fig. 27 | Adsorption energetics and configurations of H_2O on SnO_2-based catalysts. Calculated adsorption energies of H_2O on pristine and heteroatom-doped SnO_2 surfaces (left), along with corresponding optimized adsorption configurations (right).

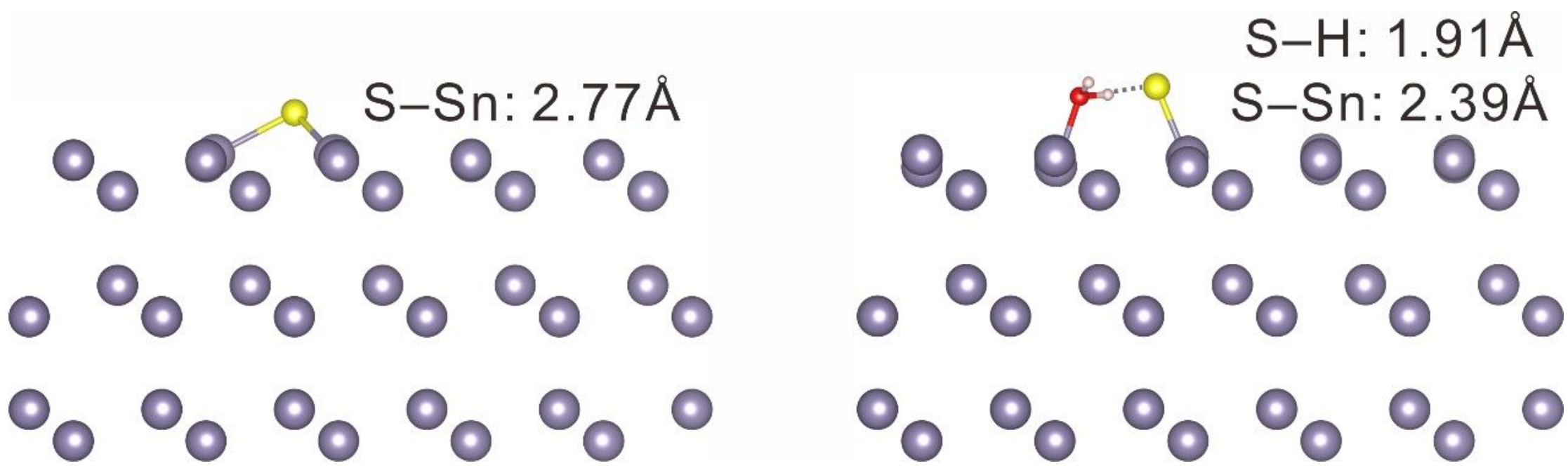

Supplementary Fig. 28 | Structural evidence for H_2O activation on S-SnO_2. Optimized geometries illustrate the interaction between interfacial H_2O and the sulfur-doped SnO_2 surface. Upon H_2O adsorption and partial dissociation, the S-Sn bond distance decreases from 2.77 Å to 2.39 Å, accompanied by the formation of an S-H bond (1.91 Å).

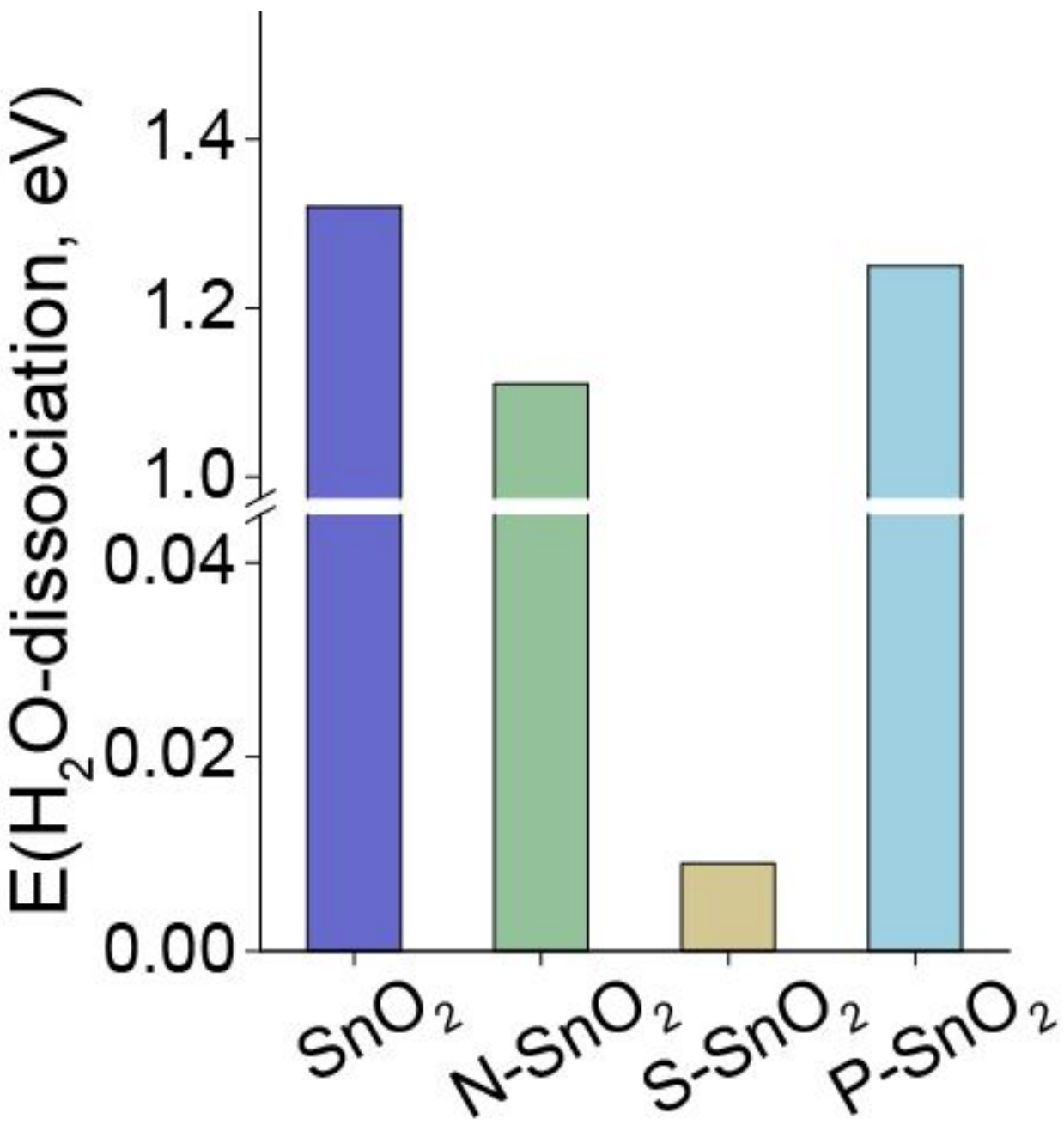

Supplementary Fig. 29 | Calculated H_2O dissociation energies on the pristine and heteroatom-doped SnO_2 surfaces.

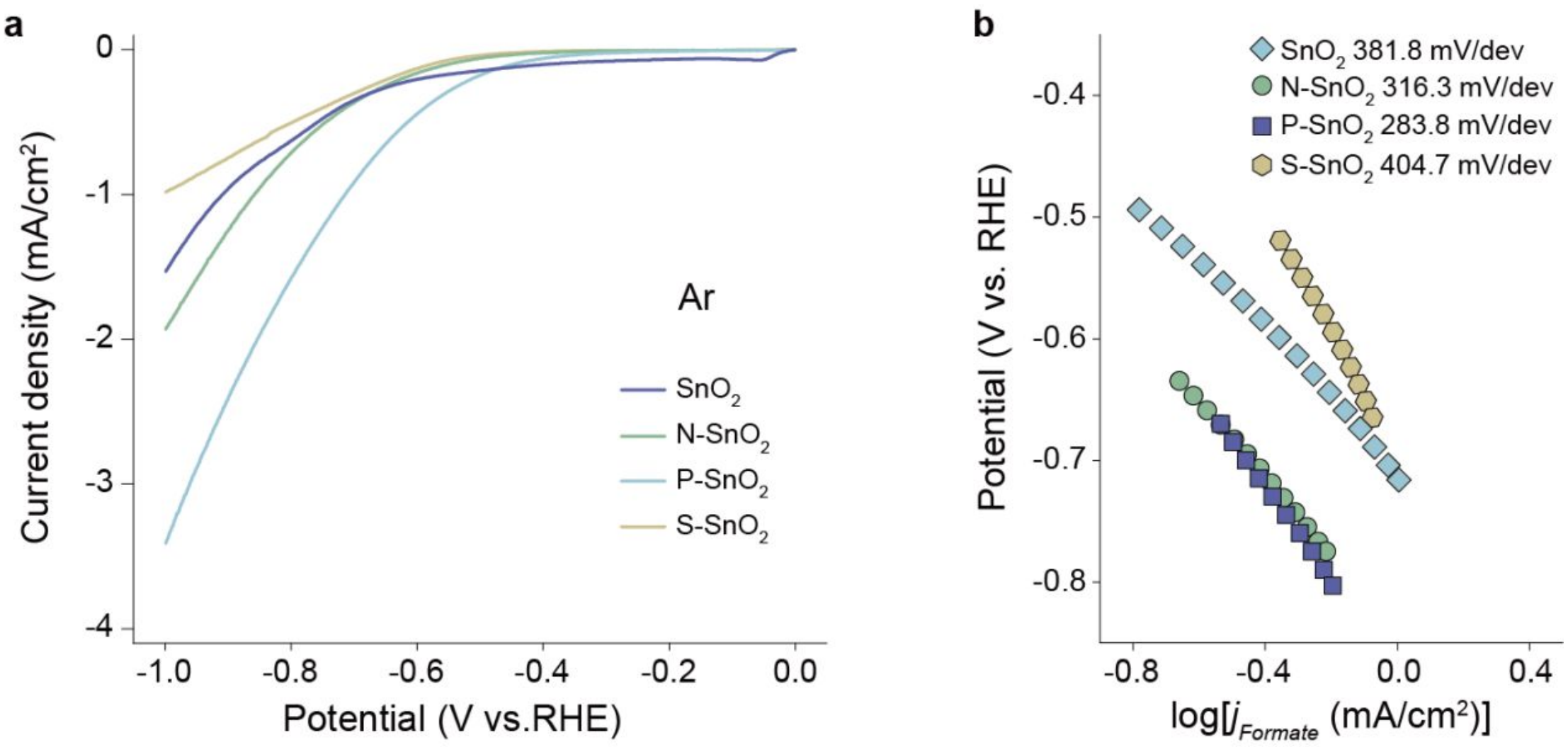

Supplementary Fig. 30 | Electrochemical kinetics analysis of SnO_2-based catalysts. (a) Linear sweep voltammetry (LSV) curves recorded under Ar-saturated electrolyte for SnO_2, N-SnO_2, P-SnO_2, and S-SnO_2. (b) Corresponding Tafel plots derived from partial current densities toward formate production.

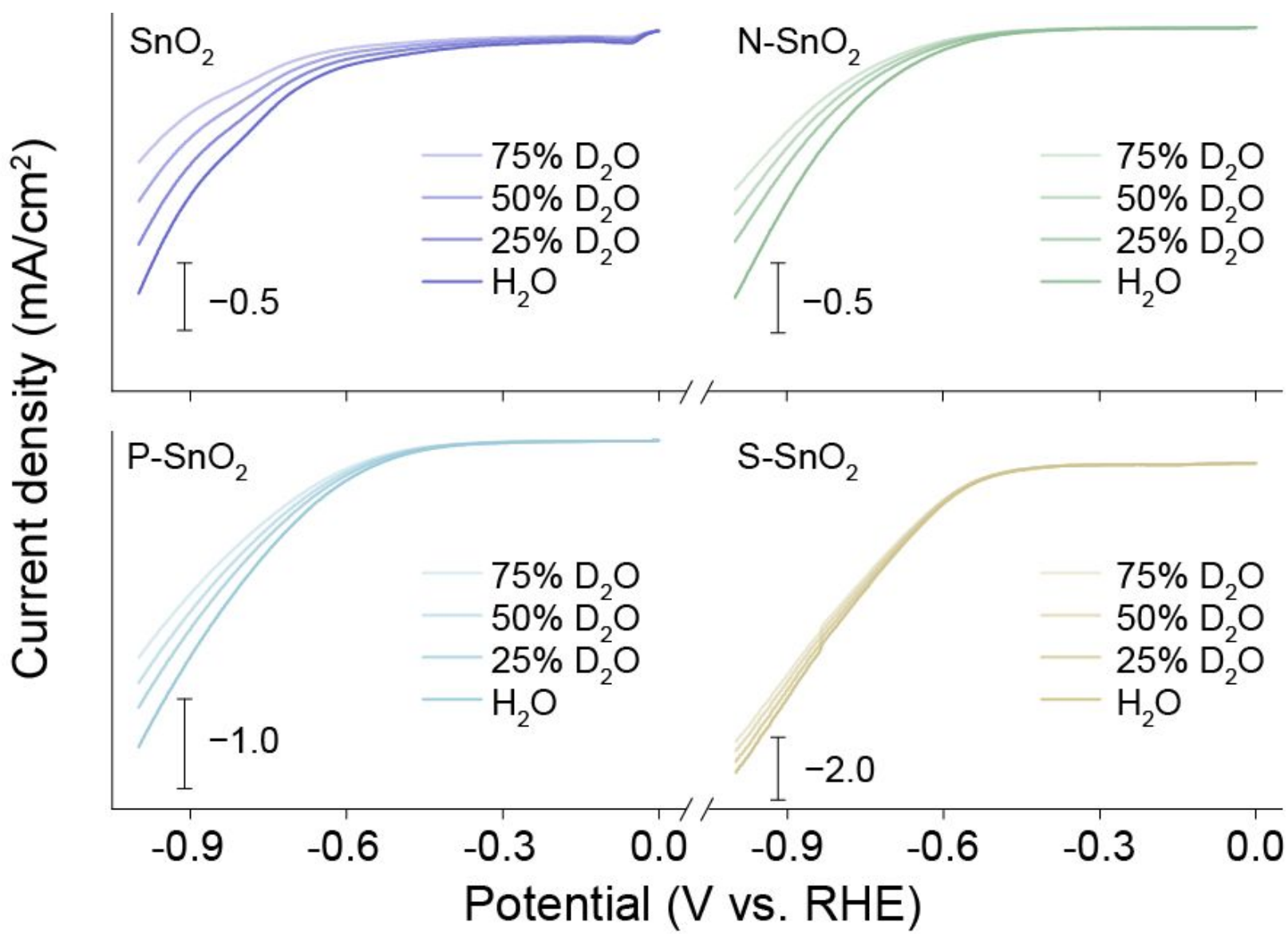

Supplementary Fig. 31 | Kinetic isotope effect (KIE) analysis using H_2O/D_2O electrolytes. Linear sweep voltammetry curves recorded in electrolytes containing varying ratios of H_2O and D_2O for SnO_2, N-SnO_2, P-SnO_2, and S-SnO_2.

Reference

(1) Kresse, G.; Furthmüller, J. Efficiency of ab-initio Total Energy Calculations for Metals and Semiconductors using a Plane-wave Basis Set. *Comp. Mater. Sci.* **1996,** *6* (1), 15-50.

(2) Blöchl, P. E. Projector Augmented-wave Method. *Phys. Rev. B* **1994,** *50* (24), 17953-17979.

(3) Lan, H.; Wang, L.; He, R.; Huang, S.; Yu, J.; Guo, J.; Luo, J.; Li, Y.; Zhang, J.; Lin, J.; Zhang, S.; Zeng, M.; Fu, L. 2D Quasi-layered Material with Domino Structure. *Nat. Commun.* **2023,** *14* (1), 7225.
